# An Oomycete CRN Effector Reprograms Expression of Plant *HSP* Genes by Targeting their Promoters

**DOI:** 10.1371/journal.ppat.1005348

**Published:** 2015-12-29

**Authors:** Tianqiao Song, Zhenchuan Ma, Danyu Shen, Qi Li, Wanlin Li, Liming Su, Tingyue Ye, Meixiang Zhang, Yuanchao Wang, Daolong Dou

**Affiliations:** Department of Plant Pathology, Nanjing Agricultural University, Nanjing, Jiangsu, China; Scottish Crop Research Institute, UNITED KINGDOM

## Abstract

Oomycete pathogens produce a large number of CRN effectors to manipulate plant immune responses and promote infection. However, their functional mechanisms are largely unknown. Here, we identified a *Phytophthora sojae* CRN effector PsCRN108 which contains a putative DNA-binding helix-hairpin-helix (HhH) motif and acts in the plant cell nucleus. Silencing of the *PsCRN108* gene reduced *P*. *sojae* virulence to soybean, while expression of the gene in *Nicotiana benthamiana* and *Arabidopsis thaliana* enhanced plant susceptibility to *P*. *capsici*. Moreover, PsCRN108 could inhibit expression of *HSP* genes in *A*. *thaliana*, *N*. *benthamiana* and soybean. Both the HhH motif and nuclear localization signal of this effector were required for its contribution to virulence and its suppression of *HSP* gene expression. Furthermore, we found that PsCRN108 targeted *HSP* promoters in an HSE- and HhH motif-dependent manner. PsCRN108 could inhibit the association of the HSE with the plant heat shock transcription factor AtHsfA1a, which initializes *HSP* gene expression in response to stress. Therefore, our data support a role for PsCRN108 as a nucleomodulin in down-regulating the expression of plant defense-related genes by directly targeting specific plant promoters.

## Author Summary

*Phytophthora* is a genus of plant-damaging oomycetes and contains over 100 species, most of which can cause enormous economic losses on crops and environmental damage to natural ecosystems. *Phytophthora* pathogens produce hundreds of effectors that act inside host cells during interaction with their host organisms. Some of these effectors may target distinct host proteins to suppress host defense and promote infection. In this study we discovered a *P*. *sojae* effector PsCRN108 that suppresses plant basal defense and reprograms expression of many host genes. Among them, ~45% of total *Heat Shock Protein* (*HSP*) genes were down-regulated. Using a GUS expression assay, we found that the effector suppressed the induction of genes driven by the *AtHSP90*.*1* promoter or a synthetic Heat Shock Element (HSE). We found that PsCRN108 preferentially targets conserved HSE’s, and can interfere with their binding to a plant heat shock transcription factor AtHsfA1a. Thus, we conclude that the effector directly targets host DNA, which is a novel mechanism by which *Phytophthora* pathogens overcome host defense responses.

## Introduction

Filamentous pathogens produce a large number of host intracellular effectors to suppress host immune responses and facilitate colonization [1–3]. In oomycete pathogens, two groups of intracellular effectors (RxLR, Arg, any amino acid, Leu, Arg; CRN, Crinkler or crinkling- and necrosis-inducing protein) have been identified [3–6]. However, their biochemical activities and molecular mechanisms are incompletely understood. By identifying host targets, some oomycete effectors have been shown to target important intracellular processes controlling disease resistance. For example, *P*. *infestans* PiAVR3a manipulates plant programmed cell death (PCD) by interacting with and stabilizing the host E3 ligase CMPG1 [7], and *P*. *infestans* PITG_03192 targets plant NAC transcriptional factors to prevent their accumulation in the host nucleus [8]. In addition, we recently found that two effectors (PsIsc1 and PsAvr3b) in *P*. *sojae* act as enzymes to suppress accumulation of the essential defense compounds salicylic acid and H_2_O_2_ [9,10].

Several effectors in bacterial plant pathogens have been identified as ‘nucleomodulins’ [11]. Among these, *Agrobacterium* VirD2 was the first described bacterial effector and may mediate integration of mobile single-stranded DNA (T-DNA) into the host genome by binding to host importin alpha [12]. The bacterial nucleomodulins may benefit pathogens by targeting host chromatin or its regulatory factors, modifying nuclear regulators, disrupting the DNA integrity or affecting epigenetic regulation [13–16]. Transcription Activator-Like (TAL) effectors are well-known nucleomodulins produced by *Xanthomonas* species and secreted via the type III secretion apparatus (T3SS) [17,18]. TAL effectors bind to TAL-specific DNA sequences and manipulate gene expression in host cells. For example, *X*. *campestris* AvrBs3 increases host susceptibility by binding directly to the plant *upa20* promoter and inducing host cell hypertrophy [19], although this mechanism is exploited by the plant resistance gene *Bs*3 whose transcription is stimulated by AvrBs3, triggering host hypersensitive responses [20]. Therefore, manipulation of the nuclear compartment is likely a strategy used by multiple pathogen species, rendering the nuclei of host cells a major battlefield in pathogen-host interactions.

Some oomycete effectors also act inside host cell nuclei. An RxLR effector from *H*. *arabidopsidis* (HaRxL44) localizes to the nucleus of plant cells and interacts with the host mediator subunit MED19a, an important regulator of gene transcription, to reduce expression of genes regulated by SA [21]. Bioinformatics and localization studies have showed that a number of oomycete CRN effectors target host cell nuclei to cause cell death and/or mediate virulence [6,22]. CRN effectors were initially identified based on their cell death-inducing activity in plants [23]. They form highly expanded gene families in oomycete genomes and diversify via gene duplication and chimeric recombination [24,25]. The functional mechanisms of only three CRN effectors have been discovered to date. The *P*. *infestans* effector CRN8, which has high sequence similarity with serine/threonine kinases, may suppress plant defense and cause cell death [26]. *P*. *sojae* effector pair, CRN63 and CRN115, manipulate host H_2_O_2_ homeostasis and promote pathogenicity by interacting with plant catalases [27]. All three CRN effectors localize to plant cell nuclei to exert their functions [26,27]. This study focuses on another *P*. *sojae* CRN effector that reduces expression of plant heat shock protein (Hsp)-encoding genes by directly targeting their promoters.

Hsp’s are widely conserved in eukaryotic organisms and play diverse roles in maintaining normal cellular functions by preventing mis-folding and aggregation of nascent polypeptides and/or by facilitating protein folding [28,29]. In mammalian cells, Hsp’s exhibit dual roles in immune responses; *i*.*e*., functioning as danger-associated molecular patterns (DAMPs) to stimulate innate immunity or participating in induction of acquired immune responses [29,30]. They also play critical roles in the innate immune systems of plants. *Arabidopsis thaliana* encodes a large number of Hsp’s of the Hsp20, Hsp70, Hsp90 and Hsp100 protein families [31]. Hsp90 complexes regulate disease resistance directly by assisting in R protein-mediated disease resistance in various plants [32]. Rice Hsp90 and its chaperone complex are critical for defense against the rice blast fungus through interaction with a chitin receptor (OsCERK1), suggesting their involvement in the PTI pathway [33]. Hsp90s also participate in *P*. *infestans* INF1- or Avr3a-triggered immune responses and are important for non-host resistance of *N*. *benthamiana* to *P*. *infestans* [34,35]. Hsp70 is required for plant defense against divergent pathogens [36]. The *Pseudomonas syringae* effector (HopI1) might directly target Hsp70 to interfere with plant immunity [37].

The expression of *HSP* genes in plant cells is finely tuned by binding of heat shock transcription factors (Hsf’s) to heat shock elements (HSE’s; 5′-GAAnnTTC-3′), which are conserved in the promoter regions of *HSP* genes [38]. The genome of *A*. *thaliana* encodes 21 Hsf’s, which can be assigned to 3 classes (HsfA, HsfB and HsfC) and 14 groups (HsfA1-9, HsfB1-4 and HsfC1). The Hsf’s contain several highly conserved functional regions, including a DNA binding domain, oligomerization domain, nuclear localization signal (NLS), C-terminal activation domains and a nuclear export signal [38,39]. During heat or other stresses, free cytoplasmic Hsf’s may oligomerize in dimers or trimers, translocate to the nucleus, undergo phosphorylation and then bind to HSE’s to activate expression of *HSP* genes [38,40]. Thus, plant Hsf’s form a complex regulatory network that mediates the flexible responses of plants to both biotic and abiotic stresses.

Here, we describe PsCRN108, a novel nucleomodulin effector from *P*. *sojae*, and show that it contributes to virulence in *P*. *sojae*. The effector contains a predicted HhH (helix-hairpin-helix) motif that is required for interaction of PsCRN108 with plant *HSP* promoters. We found that this interaction inhibited associations between *HSP* promoters and a plant heat shock transcription factor, thereby suppressing plant *HSP* gene expression and enhancing plant susceptibility to *Phytophthora* pathogens. Our findings reveal a novel mechanism used by a *Phytophthora* effector, namely reprogramming host gene expression by directly targeting to host DNA.

## Results

### PsCRN108 contains a predicted HhH motif and is expressed during infection

The *P*. *sojae* genome encodes 202 CRN effectors [25]. By sequence analysis, we found that three of them, PsCRN108, PsCRN112 and PsCRN114, were predicted to contain an HhH motif (S1A and S1B Fig). HhH motifs have DNA-binding activities [41]. Although the three PsCRN112 copies (PsCRN112a, b and c) are nearly identical to PsCRN108, specific primers could be identified within a variable region near the middle of their encoding genes (S2 Fig). Using these primers, we determined the expression patterns of *PsCRN108*, *PsCRN112a/b/c* and *PsCRN114* in different infection stages using qRT-PCR. *PsCRN108* was expressed at a high level during the early stages of infection, especially at 2 hpi, while the transcript levels of *PsCRN112a/b/c* and *PsCRN114* were low during all stages (S1C Fig). The PsCRN108 and PsCRN112 proteins contain a conserved N-terminal host translocation domain made up of a signal peptide, an LFLAK motif and a DWL domain [6], together with a C-terminal functional DC domain [25] and a nuclear localization signal (NLS) (S1A and S2 Figs). Thus, based on its high expression level during early infection, we selected PsCRN108 for further study.

### PsCRN108 is secreted from *P*. *sojae* and can enter plant cells

**Fig 1:**
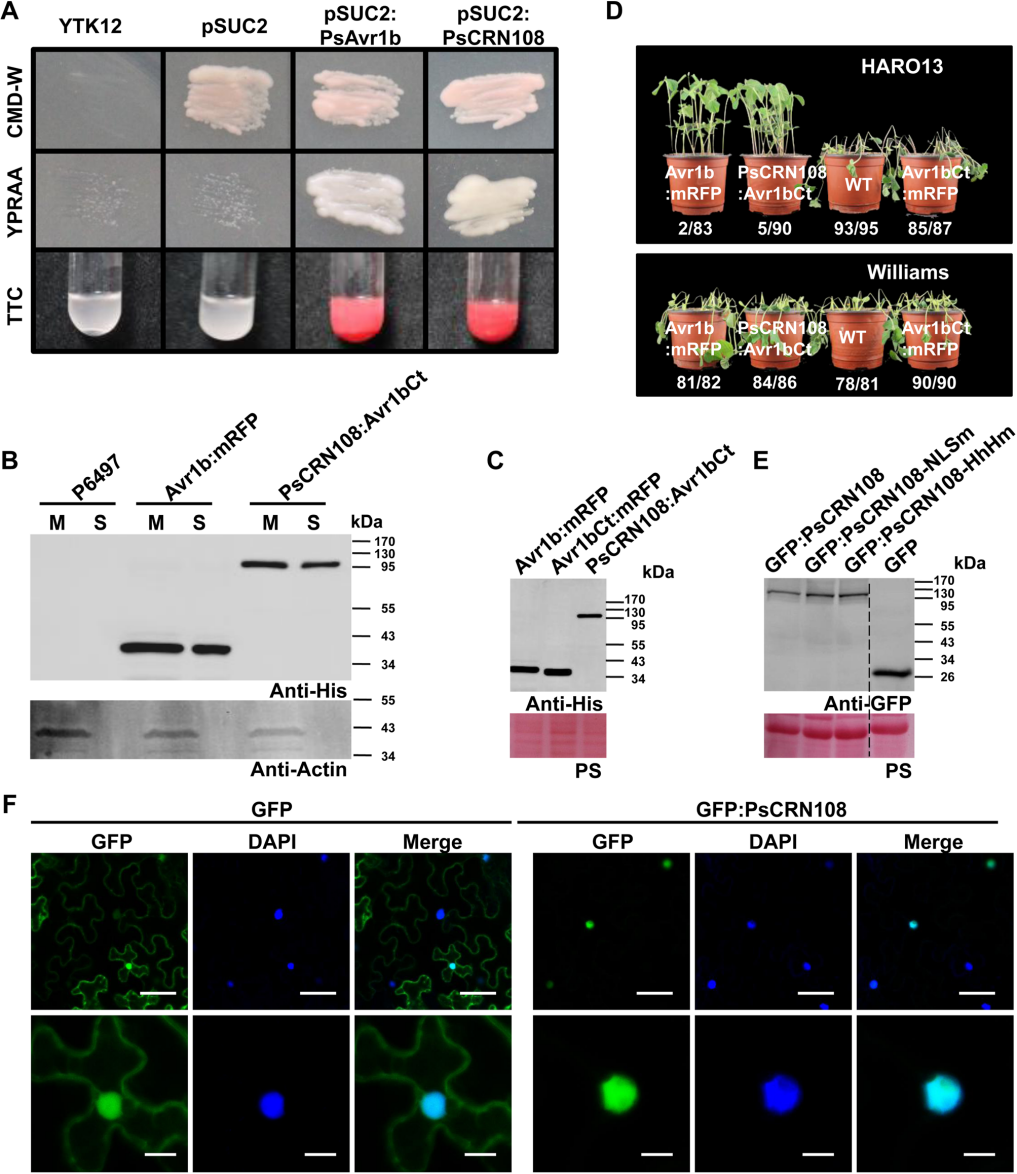
PsCRN108 is a *P*. *sojae* secreted effector that acts in the nucleus of plant cells. (A) Yeast invertase secretion assay of the predicted signal peptide of PsCRN108. The predicted signal peptide sequences and the following two amino acids (1–23) of PsAvr1b was used as the positive control to assay the predicted signal peptide sequences and the following four amino acids (1–21) of PsCRN108. CMD-W (minus Trp) plates were used to select yeast strain YTK12 carrying the pSUC2 vector. YPRAA media was used to indicate invertase secretion. An enzymatic activity test based on reduction of TTC to red-colored formazan was used to confirm invertase secretion. **(B)** Western blot analysis of proteins in culture supernatants of the *P*. *sojae* transgenic lines. PsCRN108:Avr1bCt indicates the full length of PsCRN108 fused to the Avr1b C-terminal regions (Avr1bCt aa 66–138, lacking the predicted signal peptide and RxLR host translocation domain). A *P*. *sojae* line expressing Avr1b:mRFP was used as the positive control. The His tag was attached to the C-terminus of all the fusion proteins. The non-transformed *P*. *sojae* P6497 line was used as the antibody-specificity control. Proteins extracted from mycelia (M) and culture supernatants (S) were analyzed by Western blotting using anti-His or anti-Actin antibodies. The protein sizes are expressed in kDa. (C) Western blot analysis of proteins isolated from *P*. *sojae* transgenic lines during infection. Avr1bCt:mRFP indicates Avr1b C-terminal regions fused to mRFP and is used as a control. The infected hypocotyl tissues (HARO13) including mycelia of *P*. *sojae* transgenic lines were collected at 12 hpi for protein extraction, and then Western blotting was performed using anti-His antibodies. The protein sizes are expressed in kDa. PS, Ponceau S staining. (D) Hypocotyl-inoculation assay using two soybean cultivars. Two soybean cultivars, HARO13 (*Rps1b*) and Williams (*rps*), were inoculated with the indicated *P*. *sojae* transgenic lines. Photographs were taken 2 dpi. Data (e.g. 2/83) indicate the number (2) of dead plants out of the total number (83) of plants tested. (E) Western blot analysis of GFP fusion proteins transiently expressed in *N*. *benthamiana*. The indicated proteins were extracted from leaves at 48 h after agoinfiltration and detected using anti-GFP antibodies. GFP:PsCRN108-NLSm and GFP:PsCRN108-HhHm are described in Fig 3. Dotted line indicates lanes not adjacent on the gel. The protein sizes are expressed in kDa. PS, Ponceau S staining (F) Nuclear localization of PsCRN108 in *N*. *benthamiana*. Epidermal cells of *N*. *benthamiana* leaves transiently expressing GFP alone or GFP:PsCRN108 were observed using confocal microscopy at 2 days post infiltration. DAPI staining was used to visualize the nuclei. Scale bars = 50 μm (upper panel) and 10 μm (lower panel).

Although PsCRN108 was predicted to contain a signal peptide by the SignalP3.0 HMM algorithm, the SignalP3.0 NN algorithm and the SignalP4.0 algorithms did not agree with this prediction. Therefore, to functionally validate the predicted signal peptide, we used a yeast genetic assay that is based on the requirement for secretion of invertase for yeast growth on raffinose medium [42]. The N-terminal regions of PsCRN108 (1–21 amino acids [aa]) and Avr1b (1–23 aa, positive control) were fused in-frame to yeast invertase within the vector pSUC2 [42]. Both constructs enabled the invertase mutant yeast strain YTK12 to grow on YPRAA medium and to catalyze conversion of 2, 3, 5-triphenyltetrazolium chloride (TTC) to the insoluble red-colored triphenylformazan (Fig 1A). In contrast, the negative controls (yeast strain YTK12 and YTK12 carrying the pSUC2 vector) could not grow on YPRAA plates, and TTC-treated cultures remained colorless (Fig 1A). Thus, it was concluded that PsCRN108 carries a functional secretory signal peptide.

To confirm directly whether this protein is secreted from *P*. *sojae* and also whether it is translocated into host cells during infection, we replaced the N-terminus of Avr1b (1–65 aa, including signal peptide and RxLR-dEER domain) with full-length PsCRN108 to generate the His-tagged fusion protein (PsCRN108:Avr1bCt), which was then expressed in *P*. *sojae* transformants. First, PsCRN108:Avr1bCt secretion from *P*. *sojae* was demonstrated by detecting the protein in culture supernatants of the transgenic lines by western blotting with an anti-His-tag antibody; an Avr1b:mRFP fusion line was used as a positive control and wild type (P6497) as a negative control. PsCRN108:Avr1bCt and Avr1b:mRFP proteins were detected in culture supernatants, as well as in the hyphae of the respective transformants (Fig 1B). However, the control cytoplasmic protein, actin, was found only in the hyphae (Fig 1B). The results support that PsCRN108 is secreted from *P*. *sojae*. Second, the translocation into host cells of the PsCRN108 effector was evaluated by analyzing the avirulence/virulence phenotype of *P*. *sojae* transformants on a soybean cultivar (HARO13) carrying the *Rps1b* resistance gene. Successful translocation into the cells of Rps1b plants is expected to prevent infection, as recognition of Avr1b by Rps1b inside the cell results in a successful defense response [5]. This method has previously been used successfully to determine host translocation by oomycete effectors [6,9]. The fusion proteins were expressed at the expected size in the transgenic *P*. *sojae* lines (Fig 1B) and were also detected during infection (Fig 1C). The transformants expressing PsCRN108:Avr1bCt were unable to infect hypocotyls of soybean cultivar HARO13, but could infect soybean cultivar Williams which lacks the *Rps1b* gene (Fig 1D). Similarly, the positive control line expressing Avr1b:mRFP fusion proteins [9] was avirulent on HARO13 but virulent on Williams, while the recipient WT strain (P6497) and the transgenic lines expressing Avr1bCt:mRFP (as negative controls) were virulent (Fig 1D). The results indicate that PsCRN108 could functionally replace the host cell entry domain of Avr1b. Taken together, these results indicate that PsCRN108 can be secreted from *P*. *sojae* and enter host cells during infection.

### PsCRN108 is targeted to the nuclei of plant cells

To evaluate the localization of PsCRN108 in plant cells, we used *Agrobacterium tumefaciens*–mediated transient expression assays to express GFP and GFP:PsCRN108 fusion proteins (lacking a signal peptide) in *N*. *benthamiana* (Fig 1E). In contrast to GFP alone, which was localized in both the cytoplasm and in nuclei, the GFP:PsCRN108 fusion protein was localized predominantly in nuclei, which were confirmed by DAPI staining (Fig 1F). Taken together, these findings suggest that PsCRN108 is primarily targeted to the nuclei of plant cells following translocation.

### Contribution of PsCRN108 to virulence of *P*. *sojae*

**Fig 2:**
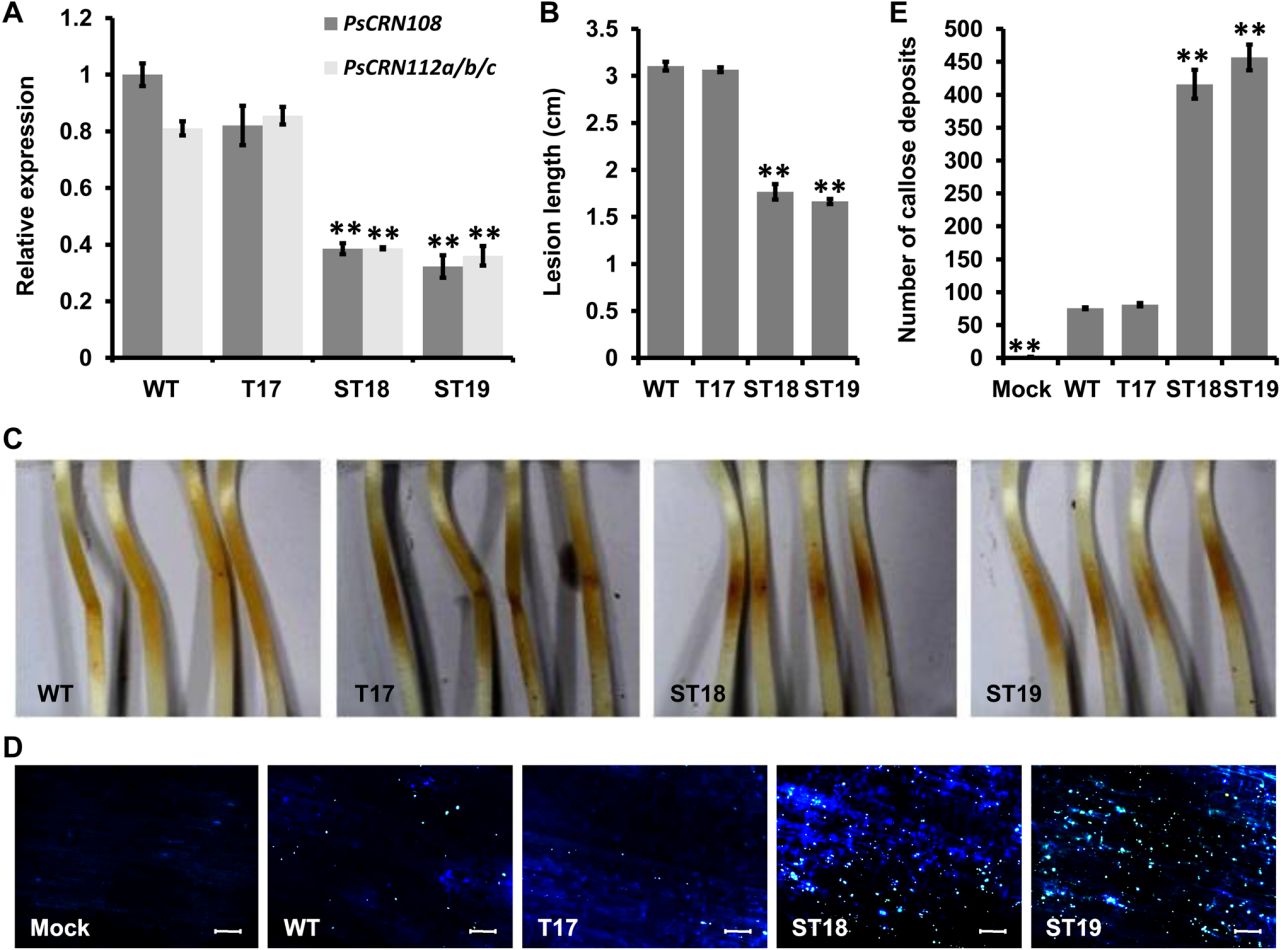
PsCRN108 contributes to the virulence of *P*. *sojae*. In all panels, WT = wild type P6497; T17 is a non-silenced transformant; ST18 and ST19 are *PsCRN108*-silenced transformants. (A) Relative transcript levels of *PsCRN108* in different transgenic *P*. *sojae* lines. The transcript levels of the *PsCRN108* and *PsCRN112a/b/c* genes were measured by qRT-PCR and normalized to those in the WT using the *actin* gene as an internal reference. Bars represent standard errors from three independent biological replicates (**, P<0.01 compared with the WT; Dunnett's test). (B) Lengths of lesions on etiolated soybean hypocotyls inoculated with *P*. *sojae*. A susceptible soybean cultivar (Williams) was inoculated with ~100 zoospores of each *P*. *sojae* line. Lesion lengths were measured at 36 hpi in three independent biological replicates, each of which comprised at least nine plants (**, P<0.01 compared with WT; Dunnett's test). (C) Phenotypes of lesions on etiolated soybean hypocotyls. Photographs representative of three independent experiments were taken at 36 hpi. (D/E) Callose deposition in etiolated soybean hypocotyl epidermal cells infected with *P*. *sojae*. Representative micrographs (**D**) of etiolated soybean hypocotyls that were inoculated with *P*. *sojae* zoospores or water (Mock) as indicated and then stained by aniline blue at 10 hpi. Bar = 50 μm. Number of callose deposits (**E**) per microscopic field was quantitated using ImageJ software. The data are mean ± SEM of numbers of callose deposits per microscopic field in three independent biological replicates, each of which comprised at least three soybean hypocotyls (**, P<0.01 compared with WT; Dunnett's test).

To determine the contribution of PsCRN108 to *P*. *sojae* virulence, gene silencing was carried out in *P*. *sojae* transgenic lines using the *PsCRN108* sequence. Based on qRT-PCR, two independent silenced transformants (ST18 and ST19) were obtained in which *PsCRN108* transcripts were reduced to <40% of the recipient WT strain (P6497). *PsCRN112* transcript levels were also reduced to less than 40% in these lines. Another transformant (T17) was selected as a negative control, because it exhibited only a slight reduction in the *PsCRN108* and *PsCRN112* transcript levels (Fig 2A). All the above transformants showed normal filamentous growth in culture (S3 Fig). Compared to the WT and T17, the two silenced lines (ST18 and ST19) showed a significant (P<0.01) reduction in virulence on soybean seedlings (Fig 2B and 2C). To determine the ability of the silenced lines to suppress host defense responses, we monitored callose deposition in infected soybean hypocotyls. The tissues challenged with silenced transformants showed significantly enhanced callose deposition compared with the WT and T17 (Fig 2D and 2E). These results together suggest that PsCRN108 contributes to *P*. *sojae* virulence by suppressing host defense responses. We also cannot rule out that PsCRN112 also contributes to virulence, despite the fact that its transcript levels are around six-fold lower than PsCRN108.

### PsCRN108 suppresses plant basal defense and promotes plant susceptibility

**Fig 3:**
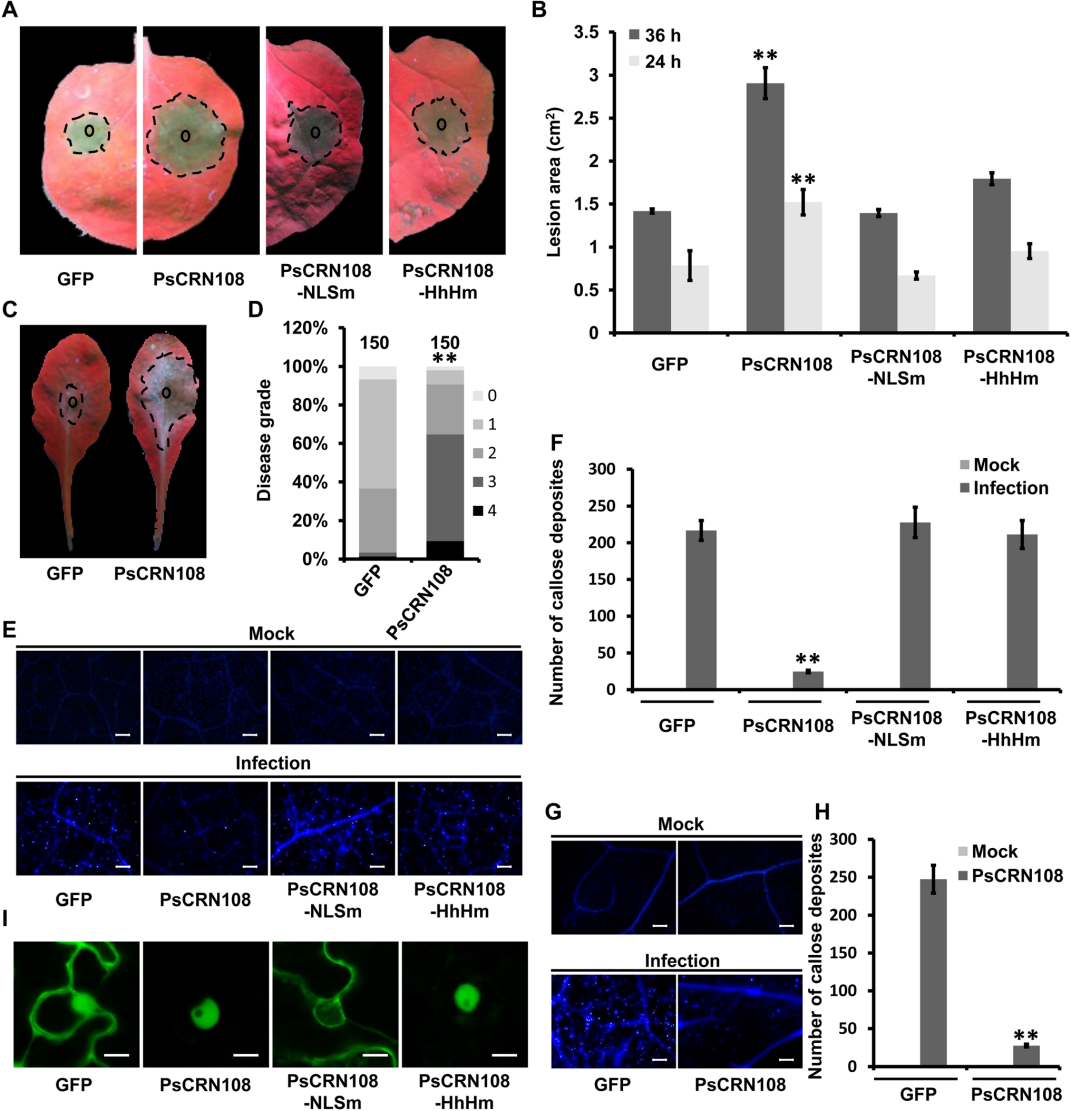
PsCRN108 suppresses defense responses in *N*. *benthamiana* and *A*. *thaliana* and promotes susceptibility. (A) Representative photographs of *N*. *benthamiana* leaves inoculated with *P*. *capsici*. The leaves were infiltrated with *A*. *tumefaciens* carrying genes encoding GFP (as a control), PsCRN108, or the indicated mutants. PsCRN108-NLSm, the substituted mutation of the predicted NLS (PsCRN108^KKRR120-123AAAA^); PsCRN108-HhHm, the substituted mutation of the predicted HhH motif (PsCRN108^L766A;G768A;V769A^). *P*. *capsici* zoospores (~100) were inoculated into the infiltrated area 48 hours after infiltration, and photographs were taken at 36 hpi. Smaller circles indicate the inoculation sites and the dotted margins indicate the lesion areas. (B) Lesion areas of inoculated *N*. *benthamiana* leaves. Lesion areas (cm^2^) were measured at 24 and 36 hpi, and calculated from three independent biological replicates using at least six leaves each (**, P<0.01 compared with GFP; Dunnett's test). (C) Representative photographs of transgenic *Arabidopsis* leaves inoculated with *P*. *capsici*. Stable transgenic *Arabidopsis* leaves expressing GFP or PsCRN108 were inoculated with ~100 *P*. *capsici* zoospores, and photographs were taken at 36 hpi. Smaller circles indicate the inoculation sites and the dotted margins indicate the lesion areas. (D) Disease rating distributions of transgenic *Arabidopsis* plant leaves. Total 150 leaves inoculated with *P*. *capsici* as in (C) were graded on a scale 0–4 as described in the Methods. **, P<0.01 for the comparison of PsCRN108 to GFP; Wilcoxon rank sum test. (E-H) Callose deposition in *P*. *capsici*-inoculated leaves. *P*. *capsici*-inoculated leaves of *N*. *benthamiana* as in (A) and of transgenic *A*. *thaliana* as in (C) were stained with aniline blue at 10 hpi to visualize callose. (E, G) Representative images of callose deposition in *N*. *benthamiana* (**E**) and *Arabidopsis* (**G**) tissues. Scale bars = 50 μm. (F, H) The numbers of callose deposits were quantified using *ImageJ* software. The data indicate the mean ± SEM numbers of callose deposits per microscopic field in three independent biological replicates, each of which comprised at least three leaves and four fields were counted per leaf (**, P<0.01 compared with GFP, Dunnett's test in *N*. *benthamiana* (**F**); **, P<0.01, *t* test in *A*. *thaliana* (H)). (I) Confocal imaging of non-inoculated *N*. *benthamiana* epidermal cells expressing each of the four proteins. Photographs were taken 48 hours post-infiltration. Scale bars = 10 μm.

We characterized the mechanism of PsCRN108 action using two model plants, *N*. *benthamiana* and *A*. *thaliana*. The fusion proteins of GFP:PsCRN108 or GFP alone (negative control) were transiently expressed in *N*. *benthamiana* leaves (Fig 1E). *P*. *capsici* zoospores were then inoculated onto the infiltrated area 48-hours after infiltration, and the sizes of the resulting lesions were recorded 24 and 36 hpi. Expression of GFP:PsCRN108 in *N*. *benthamiana* enhanced *P*. *capsici* growth compared with the *GFP* control (Fig 3A and 3B). We then generated stable transgenic *Arabidopsis* plants expressing GFP:PsCRN108 (lacking the signal peptide) or GFP alone under the control of the CaMV 35S promoter. Appropriate protein sizes were confirmed by Western blot (S4A Fig) and transgenic plants expressing GFP:PsCRN108 showed no differences in plant growth compared with transgenic plants expressing GFP (S4B Fig). Fluorescence observations of GFP and DAPI staining also indicated PsCRN108 localization to the nucleus of the *Arabidopsis* cells (S4C Fig). Inoculation assays showed that the *PsCRN108*-transgenic plants were more susceptible to *P*. *capsici* infection (Fig 3C and 3D). Moreover, the callose deposition and ROS production were significantly reduced in both *N*. *benthamiana* and *A*. *thaliana* by GFP:PsCRN108 expression (Fig 3E–3H and S5 Fig). These results together suggest that PsCRN108 can suppress plant defense responses and promote susceptibility.

To test the role of specific motifs of PsCRN108 in its function, we generated two mutations of PsCRN108 by amino acid substitutions. PsCRN108-HhHm (PsCRN108^L766A;G768A;V769A^) contained mutations of the three residues required for DNA binding by HhH motifs [41,43]. PsCRN108-NLSm (PsCRN108^KKRR120-123AAAA^), contained mutations in the residues predicted to be responsible for nuclear localization. Both mutated proteins were successfully expressed (Fig 1E). Accumulation of PsCRN108 in the nucleus of *N*. *benthamiana* cells was abolished by mutation of the NLS but was unaffected by mutation of the HhH motif (Fig 3I and S6 Fig). Both PsCRN108-NLSm and PsCRN108-HhHm failed to significantly promote plant susceptibility to *P*. *capsici* infection (Fig 3A and 3B), and both failed to suppress callose deposition and ROS accumulation in *N*. *benthamiana* (Fig 3E and 3F and S5 Fig). Collectively, these data indicate that nuclear localization and the HhH motifs are both important for suppression of plant defenses by PsCRN108.

### PsCRN108 inhibits expression of plant *HSP* genes

**Fig 4:**
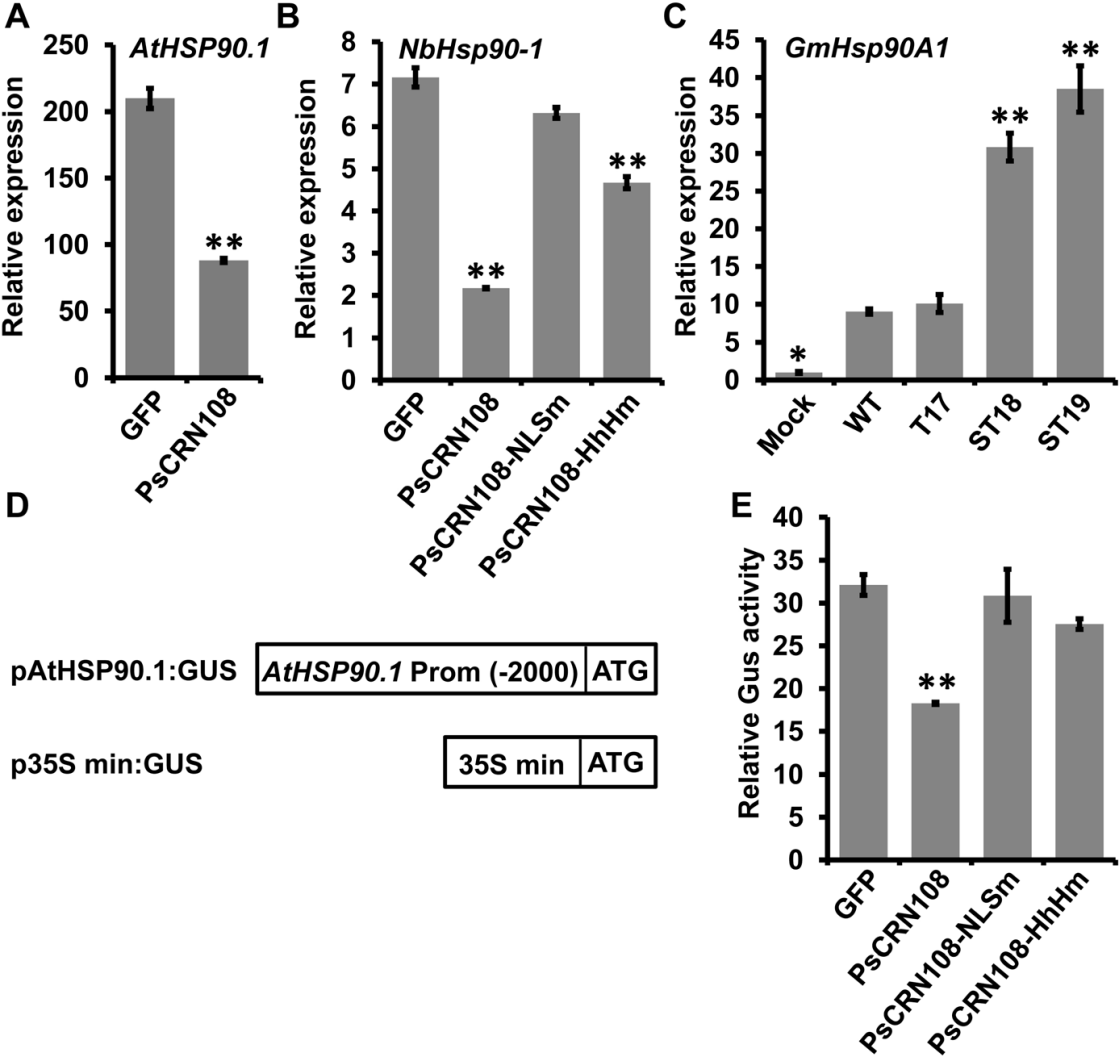
PsCRN108 interferes with plant *HSP* gene expression. (A) Relative transcript levels of *AtHSP90*.*1* in transgenic *Arabidopsis* leaves. The transcript levels of *AtHSP90*.*1* (*AT5G52640*) in stable transgenic *Arabidopsis* leaves expressing *GFP* or *PsCRN108* were measured by qRT-PCR using RNA samples extracted from *P*. *capsici* inoculated leaves (10 hpi) and mock-treated leaves. The relative transcript levels during infection were normalized to those of mock-treated leaves expressing *GFP* using *Actin2* as an internal reference. Values are means ± SEM of three independent biological replicates (**, P<0.01; *t*-test). (B) Relative transcript levels of *NbHsp90-1*. The transcript levels of *NbHsp90-1* in *N*. *benthamiana* leaves expressing *GFP*, *PsCRN108* or the indicated *PsCRN108* variants were measured by qRT-PCR using RNA samples extracted from *P*. *capsici* inoculated leaves (10 hpi) and mock-treated leaves. The relative transcript levels during infection were normalized to those of mock-treated leaves expressing GFP using *NbEF1α* gene as an internal reference. Values are means ± SEM of three independent biological replicates (**, P<0.01 compared with GFP; Dunnett's test). (C) Relative transcript levels of *GmHsp90A1*. Hypocotyls of etiolated soybean cultivar (Williams) were challenged with zoospores of the indicated *P*. *sojae* lines, and RNA was extracted at 10 hpi. The transcript levels of *GmHsp90A1* in soybean inoculated with transgenic *P*. *sojae* lines were normalized to levels of those of mock-treated soybean using *ACT20* gene as an internal reference. Values are the means ± SEM of three independent biological replicates (**, P<0.01; *, P<0.05 compared with the WT; Dunnett's test). (D) Schematic of the constructs used in the GUS activity assay. A -2000 bp promoter region of *AtHSP90*.*1* were fused to *GUS*. “ATG” is the translational start site for the *GUS* gene. The 35S min promoter fused to *GUS* (p35S min:GUS) was used for normalization of the relative GUS activity. (E) Relative GUS enzyme activity in *N*. *benthamiana*. The constructs of pAtHSP90.1:GUS and p35S min:GUS were transiently introduced into *N*. *benthamiana* leaves together with constructs encoding GFP, PsCRN108 or the indicated PsCRN108 variants. The leaves were immersed in a solution containing *P*. *capsici* zoospores 48 h post infiltration, and the protein was extracted at 3 hpi. GUS activity levels were measured using a fluorometric assay. Each column represents the ratio from the pAtHSP90.1:GUS construct relative to that from the p35S min:GUS construct. Values are the mean ± SEM of three independent biological replicates with three technological replicates each (**, P<0.01 compared with GFP; Dunnett's test).

To investigate the mechanisms underlying suppression of plant defense by PsCRN108, we used Illumina-based RNAseq assays to compare differential gene expression levels between *PsCRN108*- and *GFP*-transgenic *Arabidopsis* lines. In *P*. *capsici*-infected *Arabidopsis* plants expressing GFP:PsCRN108, 98 transcripts were more than 1.5-fold elevated and 211 were more than 1.5-fold reduced (q < 0.05) compared to infected *GFP*-transgenic plants (S1 Table). Interestingly, functional annotations revealed that 23 *HSP* genes were down-regulated, accounting for ~45% of the 51 *HSP* genes that have been identified in *Arabidopsis* (S2 Table). In the *PsCRN108*-transgenic line, aggregate transcript levels of all the *HSP* genes were around half those in the *GFP*-transgenic line. Moreover, among the 14 most highly expressed *HSP* genes in the control GFP-expressing lines (FPKM > 200), 11 were significantly reduced (q<0.05) in the *PsCRN108*-expressing line (S2 Table). To validate the sequencing results, we selected 7 representative *HSP* genes for qRT-PCR analysis. The 7 genes comprised two HSP20s, two HSP70s, two HSP90s and one HSP101; based on the RNAseq results all 7 were highly expressed in the control and significantly down-regulated by PsCRN108 (S2 Table). qRT-PCR assay of the 7 genes revealed that their transcript levels were elevated by pathogen infection, but were significantly (P<0.01) reduced in the *PsCRN108*- transgenic line compared with the *GFP*- transgenic line both with and without pathogen infection (Table 1 and Fig 4A). The results suggest that PsCRN108 suppresses transcription of *HSP* genes.

**Table 1:** Transcriptional levels of *HSP* genes in transgenic *Arabidopsis*.

Gene ID and HSP family	MT (Genes/ Actin2)^a^			PI (Genes/ Actin2)^a^			RNAseq (PI)^c^		
	GFP (10^−3^)	108 (10^−3^)	FC^b^	GFP (10^−3^)	108 (10^−3^)	FC^b^	GFP	108	FC^b^
*AT1G53540*, AtHSP20	0.28	0.18	-1.56	140	80.5	-1.74	710	340	-2.09
*AT2G29500*, AtHSP20	2.37	1.13	-2.10	217	138	-1.57	689	288	-2.39
*AT3G12580*, AtHSP70	1.44	1.13	-1.27	230	61.6	-3.74	778	403	-1.93
*AT1G16030*, AtHSP70	1.06	0.85	-1.25	69.7	31.2	-2.24	250	116	-2.15
*AT5G56030*, AtHSP90	59.9	31.2	-1.92	908	397	-.2.29	229	136	-1.64
*AT5G52640*, AtHSP90	6.79	3.36	-2.02	1424	598	-2.38	428	214	-2.00
*AT1G74310*, AtHSP101	2.06	1.28	-1.61	132	62.4	-2.11	364	173	-2.10

Notes:

^a^ qRT-PCR was performed to verify the transcript levels of *HSP* genes in transgenic *Arabidopsis* plants (GFP, *GFP*- transgenic line; 108, *PsCRN108*- transgenic line). The mock-treated (MT) plants or pathogen-infected at 10 hpi (PI) plants were used. *HSP* transcript levels were normalized to that of the constitutively expressed *Actin2* (*At3G18780*) gene using the equation 2^(Ct^[Actin2]^-Ct^[GENE]^). Data (in 10^−3^) are means from three biological replicates.

^b^ Fold change (FC) represents the transcript levels of *HSP* genes in *PsCRN108*-transgenic *Arabidopsis* relative to those in *GFP*-transgenic *Arabidopsis*. ‘–’ indicates down-regulation in *PsCRN108*- transgenic line.

^c^ Data represent FPKM (fragments per kilobase of exon per million fragments mapped) value based on RNAseq.

Among the 4 *HSP* gene families, HSP90’s have been shown to have a direct involvement in plant defense [32–35]. To further confirm PsCRN108-mediated suppression of *HSP90* expression, we tested the effect of PsCRN108 on the expression of the *NbHsp90-1* gene in *N*. *benthamiana* and of *GmHsp90A1* in soybean, respectively. After *P*. *capsici* infection, *N*. *benthamiana* expressing GFP:PsCRN108 showed lower *NbHsp90-1* gene transcript levels compared with *N*. *benthamiana* expressing GFP (Fig 4B). In the case of *GmHsp90A1*, we compared the transcript levels of the gene in soybean infected with *P*. *sojae* WT, T17 or *PsCRN108*-silenced lines (ST18 and ST19). As shown in Fig 4C, soybean plants infected with ST18 and ST19 showed markedly higher *GmHsp90-A1* transcript levels compared with plants infected with WT and T17, indicating that silencing of *PsCRN108* in *P*. *sojae* may reduce its ability to suppress host *GmHsp90-A1* gene transcription. Collectively, the results suggest that PsCRN108 can suppress transcription of *HSP* genes in *Arabidopsis*, *N*. *benthamiana* and soybean.

### The nuclear localization and HhH motifs are required for suppression of plant *HSP* gene transcription by PsCRN108

We next determined the contributions of its nuclear localization and HhH motifs to the suppression of *HSP* gene transcription by PsCRN108. The *NbHsp90-1* transcript levels in *N*. *benthamiana* transiently expressing GFP, GFP:PsCRN108, GFP:PsCRN108-NLSm or GFP:PsCRN108-HhHm (all without signal peptides) were compared by qRT-PCR. GFP:PsCRN108-NLSm did not cause significant suppression (p>0.05) of *NbHsp90*.*1* transcripts compared to the GFP-expressing line, while transcript suppression was significantly (P<0.01) attenuated in leaves expressing GFP:PsCRN108-HhHm (Fig 4B). To more directly test the effect of PsCRN108 on Hsp90 promoter activity, we generated a construct (pAtHSP90.1:GUS) in which the *β-glucuronidase* (*GUS*) reporter gene was driven by native *AtHSP90*.*1* promoter (2 kb). The reporter construct was expressed together with GFP, PsCRN108, PsCRN108-NLSm or PsCRN108-HhHm in *N*. *benthamiana*. Based on measurement of relative GUS enzyme activity (Fig 4D and 4E), we found that PsCRN108 (P<0.01) but not GFP or the mutants (p>0.05), could significantly reduce the expression of the *GUS* gene. The results together suggest that both the nuclear localization and HhH motifs are required for suppression of *HSP* gene transcription.

### PsCRN108 targets *HSP* gene promoters

**Fig 5:**
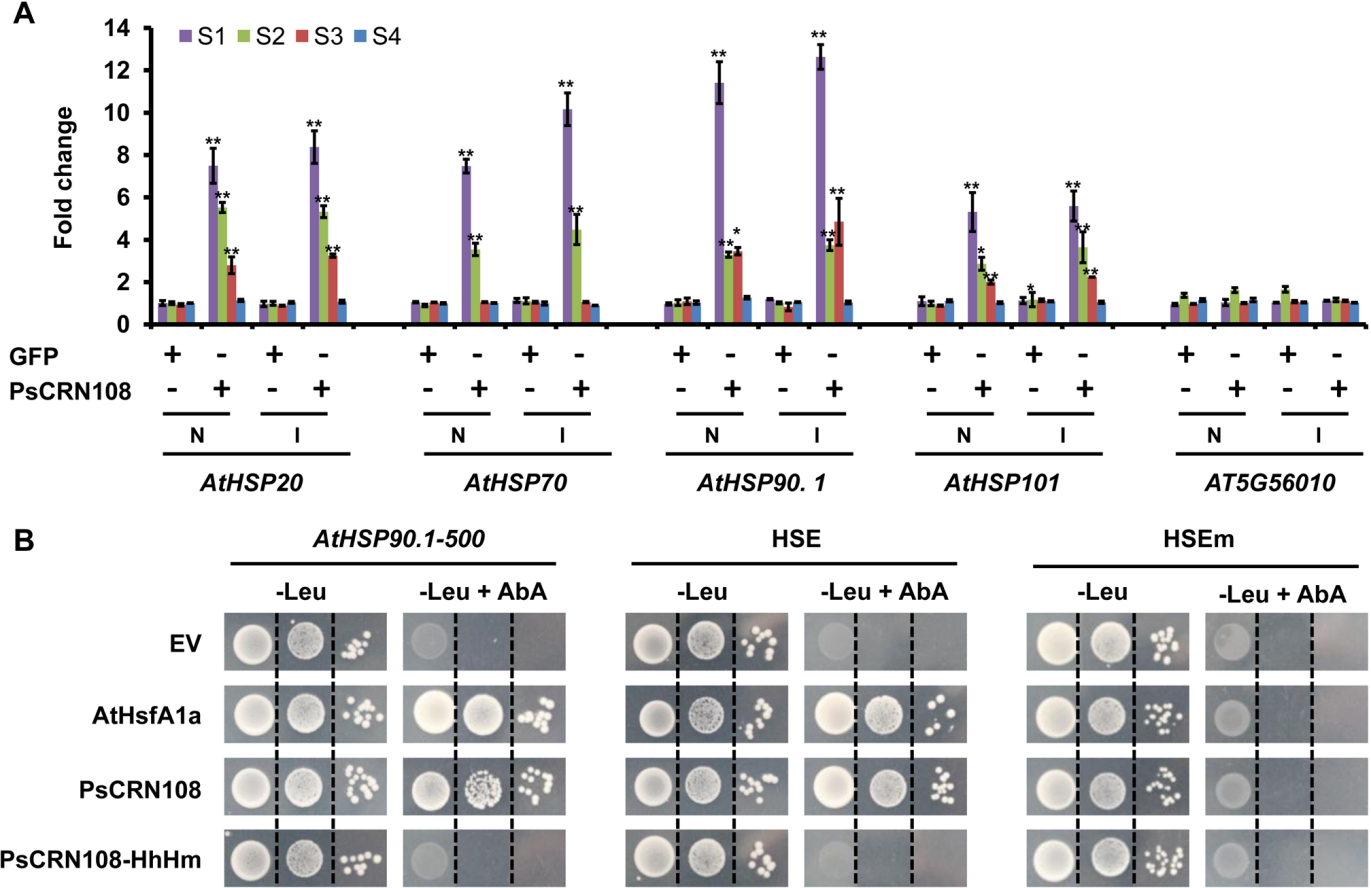
PsCRN108 interacts with *Arabidopsis HSP* gene promoters and HSE’s. (A) Enrichment of *AtHSP* gene promoters as determined by ChIP assays. Transgenic *Arabidopsis* plants expressing GFP or GFP:PsCRN108 infected with *P*. *capsici* at 10 hpi (I) or without (N) infection were used for ChIP assays. Specific primers were designed for four fragments (S1-4 are described in S7 Fig) in the five selected *Arabidopsis HSP* promoters (*AtHSP20*, *AT1G53540*; *AtHSP70*, *AT3G12580*; *AtHSP90*, *AT5G52640*; *AtHSP101*, *AT1G74310*; and *AT5G56010*, another *HSP90* gene used as a negative control). qPCR analysis was performed on immunoprecipitated DNA using anti-GFP antibodies. Values for the ChIP samples were firstly normalized to the input control and then divided by the no-antibody control to obtain the fold enrichment values. Values are the means ± SEM of three independent biological replicates (**, P<0.01; *, P<0.05 compared with GFP without infection; Dunnett’s test). (B) Yeast one-hybrid (Y1H) assay of PsCRN108 with the *AtHSP90*.*1* promoter region and HSE. A 500-bp (-1 to -500) fragment of the *AtHSP90*.*1* promoter, a 19 bp HSE (CCAGAAGCTTCCAGAAGCC), or a 19 bp HSE mutant (HSEm, CCAtAAGCTTaCAtAAGCC) were integrated into the genome of yeast upstream of the *AUR1-C* gene to produce yeast bait strains. The lowest concentrations of Aureobasidin A (AbA) that limited the growth of yeast bait strains were determined before transformation with the pGAD plasmid (*AtHSP90*.*1*–500, 200 ng ml^-1^; HSE, 500 ng ml^-1^; HSEm, 500 ng ml^-1^) and were used to assess the pGAD transformants. Yeast growth on selective medium (-Leu +AbA) was recorded on day 3 as an indicator of protein—DNA interactions. pGAD:AtHsfA1a served as the positive control and pGAD as the negative control.

Since PsCRN108 appeared to suppress *AtHSP90*.*1* promoter-mediated GUS expression (Fig 4E) and contains a functionally essential DNA-binding motif (S1A and S2 Figs), we speculated that PsCRN108 might directly target *HSP* promoters. As one test of this hypothesis, we carried out chromatin immunoprecipitation (ChIP) on *PsCRN108*- transgenic *Arabidopsis* plants and *GFP*- transgenic plants with and without pathogen infection using an anti-GFP antibody. The enrichment of four independent promoter segments each of four *AtHSP* genes (*AtHSP20*, *AT1G53540*; *AtHSP70*, *AT3G12580*; *AtHSP90*.*1*, *AT5G52640*; *AtHSP101*, *AT1G74310*) in immunoprecipitated DNA was measured by qPCR, using four primer pairs to target each promoter segment (S7 Fig). There was no apparent enrichment of any segments in the *GFP*-transgenic lines with or without pathogen infection (Fig 5A). In contrast, the S1 and S2 segments of *AtHSP20* and *AtHSP70*, and the S1 segment of *AtHSP90*.*1* and *AtHSP101* were highly enriched (>5-fold) in seedlings expressing GFP:PsCRN108. Also the S3 segment of *AtHSP20*, and the S2 and S3 segments of *AtHSP90*.*1* and *AtHSP101* were moderately enriched (> 3-fold). However, the S4 segments of all *HSP* genes and all four segments of a negative gene (*AT5G56010*, an *HSP90* gene which is not significantly down-regulated by PsCRN108 and contains no HSE) were not enriched in precipitated chromatin from *PsCRN108* plants (Fig 5A). Thus, PsCRN108 binds to the chromatin of the four PsCRN108-regulated *HSP* promoters evaluated, with highest enrichment of regions closest to HSE’s.

As a second test of PsCRN108 targeting of HSP promoters, we used a yeast one-hybrid (Y1H) assay to examine a region (-1 to -500) of the *AtHSP90*.*1* promoter (*AtHSP90*.*1–500*). This fragment was highly enriched in chromatin ChIP experiments. The transcription factor (AtHsfA1a), which binds to the *HSP* promoter and HSE [44,45], was used as a positive control; and PsCRN108-HhHm was used as a negative control. *AtHSP90*.*1–500* was inserted into the yeast genome upstream of an Aureobasidin A resistance gene (*AUR1-C*) reporter to generate a *AtHSP90*.*1–500* yeast bait strain. A pGAD vector carrying PsCRN108, PsCRN108-HhHm (both lacking SP) or AtHsfA1a was then transformed into this bait strain. As shown in Fig 5B, expression of PsCRN108 and AtHsfA1a in the bait strain conferred activation of *AUR1-C* resulting in Aureobasidin A resistance, while PsCRN108-HhHm expression and an empty vector did not. These results support that PsCRN108 targets the *AtHSP90*.*1* promoter region in an HhH motif-dependent manner.

As a third test of PsCRN108 targeting of the *AtHSP90*.*1* promoter, PsCRN108, PsCRN108-HhHm and AtHsfA1a were each tagged with maltose-binding protein (MBP) and produced in *Escherichia coli* (S8A Fig). The Electrophoretic Mobility Shift Assay (EMSA) was used to assess binding of each protein to the *AtHSP90*.*1–500* DNA fragment. We observed a weak binding complex trapped in the gel wells in all the conditions tested when the Alex660-labelled *AtHSP90*.*1–500* was incubated with PsCRN108 or AtHsfA1a, but this was not observed in the MBP control and was reduced with PsCRN108-HhHm (S8B Fig) suggesting that PsCRN108 may be binding directly to the promoter fragment in a manner similar to AtHsfA1a.

### PsCRN108 binds directly to heat shock elements (HSE’s)

To examine the possibility that PsCRN108 binds directly to the heat shock element (HSE) that is the binding site for Hsf’s, we used both the Y1H and EMSA assays. All of the PsCRN108-down-regulated *Arabidopsis HSP* gene promoters contain at least one HSE (GAAnnTTCnnGAA) or its variants (e.g., TTCnnGAA, GAAnnTTC and TCCnnGAAnnTTC) [38,40,46], most of which are localized at the 3’-regions of the promoters near the translational start site (S7 Fig and S2 Table). Thus, for these experiments we used a synthetic HSE [45] plus the corresponding mutant HSEm as a negative control.

For the Y1H assays, we inserted the 19 bp synthetic HSE or its mutant control (HSEm) upstream of the CYC1 minimal promoter that drives the *AUR1-C* reporter. As shown in Fig 5B, expression of both PsCRN108 and AtHsfA1a activated HSE-*AUR1-C* to confer Aureobasidin A resistance, but the empty vector and the PsCRN108-HhHm controls did not. In contrast, neither PsCRN108 nor AtHsfA1a could activate the HSEm-*AUR1-C* control. These results demonstrate that PsCRN108 promoter-targeting in yeast is sequence-specific and requires an intact HSE. Furthermore, the sequence-specific targeting requires an intact HhH motif.

For the EMSA assays, the 19 bp HSE dsDNA fragment and its 19 bp mutant control (HSEm) were used to assess *in vitro* binding by PsCRN108 and AtHsfA1a (S8C and S8D Fig). Although binding by PsCRN108 was weaker than AtHsfA1a, and the complexes produced by both proteins were trapped in the wells of the gel, PsCRN108 bound only to the intact 19 bp HSE fragment, but not the mutant HSEm fragment. Furthermore, no binding was observed by the PsCRN108-HhHm mutant (S8C Fig). To confirm the specificity of the HSE-mediated binding detected in the EMSA assay, we performed competition experiments. The Alex660-labelled HSE probe and increasing amounts of an unlabeled competitor were co-incubated with the test proteins. The concentrations of binding complexes of PsCRN108 and AtHsfA1a were progressively reduced with the addition of increasing amounts of unlabeled competitor, consistent with specific competition of binding (S8C and S8D Fig). In contrast, no binding competition was observed when the increasing amounts of unlabeled HSEm probe were used as the competitor (S8C and S8D Fig). Taken together, the results suggest that PsCRN108 can bind the HSE directly in a relatively sequence-specific manner, and that efficient binding requires the HhH motif.

### PsCRN108-HSE interactions interfere with AtHsfA1a binding

**Fig 6:**
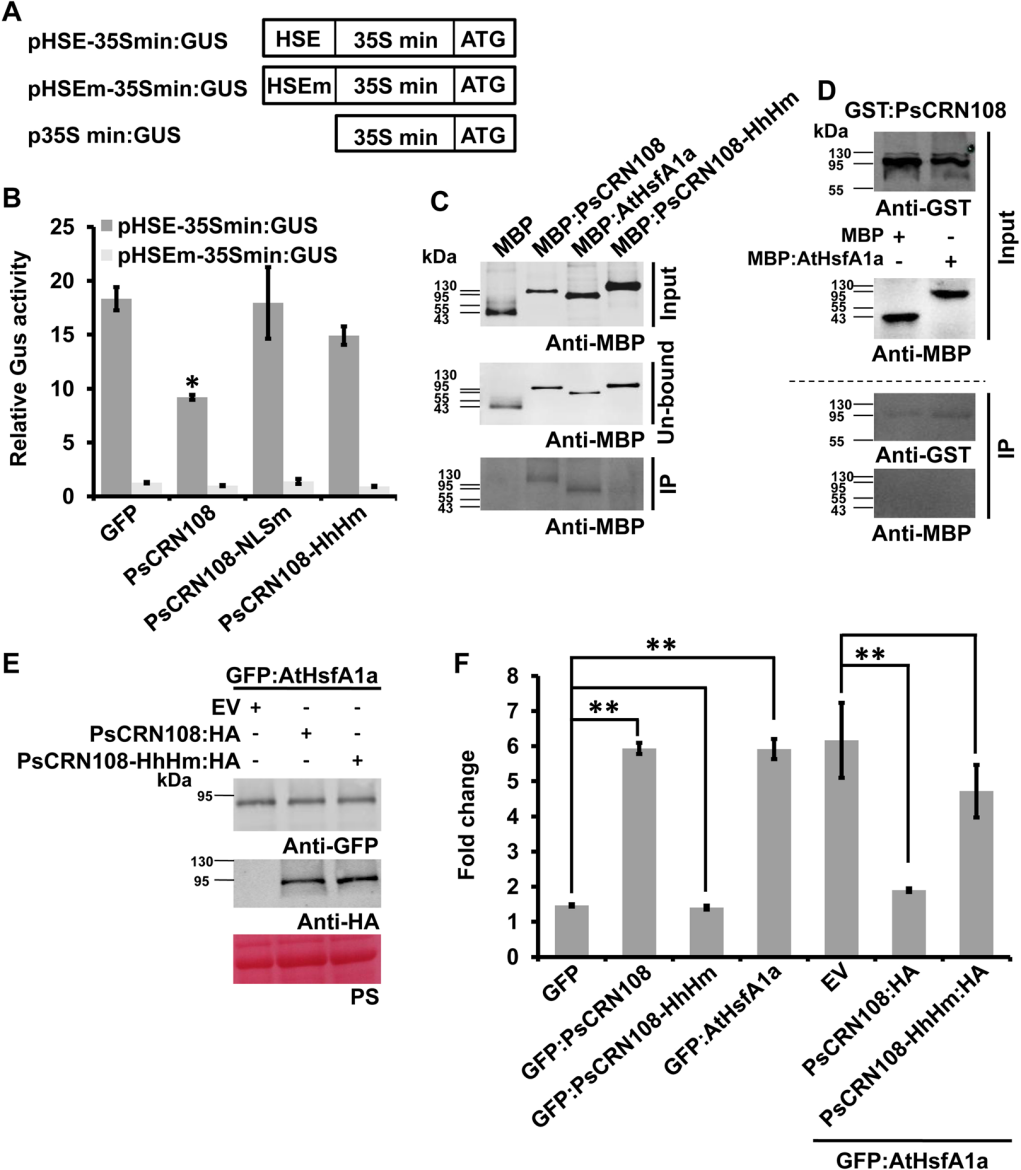
PsCRN108–HSE interaction inhibits binding to HSE of plant AtHsfA1a. (A) Schematic of the constructs used in the GUS activity assay. The HSE and HSEm were fused to the 35S minimal promoter and *GUS*. “ATG” is the translational start site for the GUS gene. The 35S minimal promoter fused to GUS was used for normalization of the relative GUS activity. (B) HSE-driven GUS activity in *N*. *benthamiana* was suppressed by PsCRN108. Leaves transiently expressing PsCRN108 or PsCRN108 mutants together with the GUS constructs were challenged with *P*. *capsici* zoospores for 3 h. Then, GUS activities were measured by fluorometric assays. The data in each column are fold changes normalized to the control (p35S min:GUS). Bars indicate standard errors from three biological replicates (*, P<0.05 compared with GFP; Dunnett’s test). (C) Pull-down of PsCRN108 by HSE. Biotinylated HSE (100 fmol) was incubated with 150 μg indicated proteins in the presence of streptavidin agarose. Upper panel, Western blot analysis of the input proteins using an anti-MBP antibody. Middle panel, Western blot detection of un-bound proteins before elution. Lower panel, proteins pulled-down by HSE. MBP and PsCRN108-HhHm were used as negative controls and AtHsfA1a as the positive control. (D) Inhibition of binding between AtHsfA1a and HSE by PsCRN108 as determined by DNA pull-down assay. Biotinylated HSE (100 fmol) was first incubated with 150 μg GST:PsCRN108 proteins (first panel) in the presence of streptavidin agarose, and then 150 μg MBP (negative control) or 150 μg MBP:AtHsfA1a (second panel) proteins were added after washing of the unbound protein. PsCRN108 (third panel), but not AtHsfA1a (fourth panel), was found to bind to HSE by Western blotting. (E) Western blot analysis of GFP- and HA- fusion proteins transiently expressed in *N*. *benthamiana* 48 h after infiltration. The protein sizes are expressed in kDa. PS, Ponceau S staining. (F) Inhibition of binding between AtHsfA1a and HSE by PsCRN108 as determined by *in vivo* ChIP assay. *N*. *benthamiana* leaves expressing the constructs as indicated were infected with *P*. *capsici* zoospores for 10 h, and then used for ChIP assays with anti-GFP antibodies. qPCR analysis was performed on immunoprecipitated DNA using primers specific for the *NbHsp90*-1 promoter (S7 Fig). Values for the ChIP samples were first normalized to the input control and then divided by the no-antibody control to obtain the fold enrichment values. Values are the means ± SEM of three independent biological replicates (**, P<0.01 compared with the indicated control; Dunnett’s test).

To further explore the mechanisms underlying the contribution of PsCRN108–HSE interactions to suppression of host *HSP* gene transcription, we determined whether PsCRN108 interferes with transcriptional activation mediated by the binding of plant endogenous transcription factors to an HSE in a *N*. *benthamiana* transient expression assay. We constructed a *GUS* reporter gene whose expression was driven by an HSE-fused minimal 35S promoter (pHSE-35Smin:GUS) or an HSEm-fused minimal 35S promoter (pHSEm-35Smin:GUS) (Fig 6A). The HSEm-fused promoter did not induce GUS expression upon *P*. *capsici* infection under any conditions. However, when *GUS* gene expression was driven by the HSE-fused promoter, the relative GUS enzymatic activity was increased by ~18-fold upon infection, when GFP was co-expressed (Fig 6B), indicating that inserted HSE may mediate induction of *HSP* expression during infection as a result of interactions with endogenous *N*. *benthamiana* transcription factors. In the presence of PsCRN108 co-expression however, the HSE-mediated increase in GUS enzymatic activity during infection was suppressed two-fold (P<0.05). In contrast, in the presence of PsCRN108-NLSm or PsCRN108-HhHm there was little or no suppression (p>0.05) of the HSE-mediated increase (Fig 6B). These results indicate that PsCRN108 can suppress HSE-mediated transcriptional activation of *HSP* genes.

To test if PsCRN108-HSE interactions could interfere with Hsf-HSE interactions *in vitro*, we determined the effects of the interactions between PsCRN108 and HSE on binding of AtHsfA1a to the same target using a competitive DNA-binding pull-down assay using *E*. *coli*-expressed proteins. First, biotinylated 19 bp HSE DNA was incubated with MBP, MBP:PsCRN108, MBP:PsCRN108-HhHm or MBP:AtHsfA1a in the presence of streptavidin agarose. After washing away the unbound proteins, only MBP:PsCRN108 and MBP:AtHsfA1a could be eluted from the streptavidin agarose (Fig 6C), which indicated that PsCRN108 could bind directly to HSE in this DNA binding assay system. Second, GST:PsCRN108 was pre-incubated with biotinylated HSE in the presence of streptavidin agarose, and then MBP or MBP:AtHsfA1a was added after washing away the unbound GST:PsCRN108 protein. MBP-AtHsfA1a was not detected in the eluted buffer suggesting that prior occupancy of the HSE by PsCRN108 could inhibit subsequent binding by AtHsfA1a (Fig 6D). Thus, we suggest that the PsCRN108-HSE interaction inhibits the binding of AtHsfA1a to HSE.

To examine the effects of PsCRN108 on the binding of AtHsfA1a to *HSP* promoters *in vivo* during infection, we performed a ChIP assay. GFP-tagged AtHsfA1a (GFP:AtHsfA1a) was co-expressed with HA-tagged PsCRN108 (PsCRN108:HA) or PsCRN108-HhHm (PsCRN108-HhHm:HA) in *N*. *benthamiana*, and then the enrichment levels of the *NbHsp90-1* promoter were determined by ChIP-qPCR assay using anti-GFP antibody. Co-expression of PsCRN108:HA or PsCRN108-HhHm:HA did not impact the expression level of GFP:AtHsfA1a (Fig 6E). As is shown in Fig 6F, expression of GFP:PsCRN108 or GFP:AtHsfA1a individually led to enrichment of the *NbHsp90-1* promoter region in the precipitated chromatin, while GFP or GFP:PsCRN108-HhHm did not produce any enrichment. These results indicated that PsCRN108 and AtHsfA1a could bind to the *NbHsp90-1* promoter *in vivo*. When PsCRN108:HA was expressed together with GFP:AtHsfA1a, enrichment of the *NbHsp90-1* promoter using the anti-GFP antibody (against GFP:AtHsfA1a) was reduced significantly (P<0.01). In contrast, when PsCRN108-HhHm:HA was used, enrichment of the *NbHsp90-1* promoter using the anti-GFP antibody was not significantly reduced (p>0.05) (Fig 6F). These data indicate that PsCRN108 can suppress the binding of a plant Hsf to an HSE *in vivo*.

### NbHsp90s are involved in plant resistance to the oomycete pathogen *P*. *capsici*

**Fig 7:**
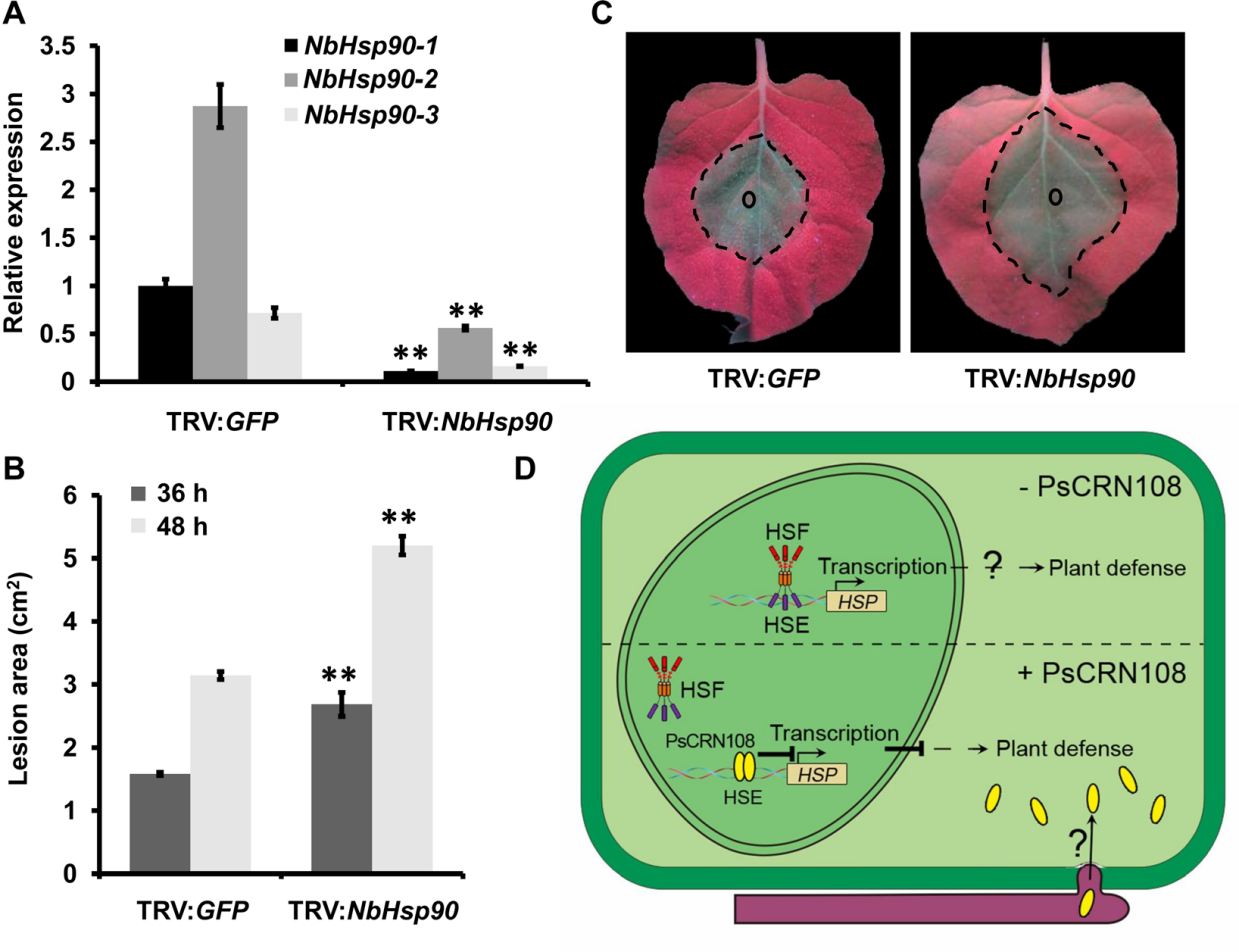
The *NbHsp90* gene family is required for *P*. *capsici* resistance in *N*. *benthamiana*. (A) Relative transcript levels of three *NbHsp90* genes. *N*. *benthamiana* leaves were infiltrated with *Agrobacterium* strains harboring the pTRV1 vector combined with pTRV2:*NbHsp90* or pTRV2:*GFP* (as a negative control). Total RNA samples were extracted 2 weeks after infiltration and subjected to qRT-PCR analysis. Transcriptional levels of three genes, *NbHsp90*-1/2/3, were normalized to the levels in *N*. *benthamiana* infiltrated with TRV:*GFP* using the *NbEF1α* gene as an internal reference. Bars represent standard errors from three independent biological replicates (**, P<0.01; *t*-test). (B) Lesion areas of TRV-*N*. *benthamiana* leaves inoculated with *P*. *capsici*. ~100 *P*. *capsici* zoospores were inoculated into the indicated silenced leaves and lesion areas were measured at 36 hpi and 48 hpi. Values (cm^2^) are the means ± SEM of three independent biological replicates, each of which comprised five leaves (**, P<0.01, *t*-test). (C) Phenotypes of TRV-infected *N*. *benthamiana* leaves inoculated with *P*. *capsici*. Representative photographs were taken at 48 h post-inoculation with *P*. *capsici* zoospores. Smaller circles indicate the inoculation sites and the dotted margins indicate the lesion areas. (D) Model of the involvement of PsCRN108 in suppression of *HSP* gene transcription and plant defense. During exposure to heat or other stresses, including biotic stresses, free cytoplasmic AtHsfA1a may translocate to the nucleus and bind as a trimer to HSE (a conserved element in the promoter regions of *HSP* genes), resulting in activation of gene expression [28,39]. *P*. *sojae* secretes the PsCRN108 effector to bind to the same binding region as AtHsfA1a in an HhH-dependent manner. The binding of PsCRN108 to HSE inhibits the association of AtHsfA1a with HSE, resulting in the suppression of *HSP* gene transcription and inhibition of plant defense responses.

It has been reported that *Hsp90* gene family is critical for non-host resistance of *N*. *benthamiana* to *P*. *infestans* [35]. To understand the impact of PsCRN108 interference with Hsp90 expression, we determined the contribution of *Hsp90* to *N*. *benthamiana* disease resistance against the native pathogen, *P*. *capsici*, by silencing *NbHsp90* genes in *N*. *benthamiana* using virus induced gene silencing (VIGS). A fragment of *NbHsp90-1*, which exhibited high sequence similarity to other members of this family, was cloned in an antisense manner into the pTRV2 vector to create a silencing construct (pTRV2-*NbHsp90*). GFP and a DNA fragment corresponding to the phytoene desaturase gene (PDS) were used as a negative control and as a marker of the effectiveness of VIGS, respectively [47]. As reported [36], abnormal and photo-bleached leaves were observed in the upper leaves of TRV:*NbHsp90*-infected plants and TRV:*PDS* infected plants, respectively (S9A Fig). Silencing of *NbHsp90-1*, *NbHsp90-2* and *NbHsp90-3* suggested that the much of the *NbHsp90* gene family was silenced (Fig 7A). *NbHsp90*-silenced plants showed enhanced growth of *P*. *capsici* on detached leaves compared with non-silenced plants, as evidenced by an almost 2-fold increase in lesion areas (Fig 7B and 7C). *NbHsp101*-silenced plants are also more susceptible to *P*. *capsici* infection compared with the non-silenced plants (S9 Fig). These results demonstrate that NbHsp90s are important for plant resistance to the oomycete pathogen *P*. *capsici*.

## Discussion

The CRN (Crinkler) and RxLR effectors are two major groups of intracellular effector proteins produced by oomycete pathogens. CRN effectors exhibit several important features. First, they represent an ancient effector family that is widely distributed in oomycete pathogens [6], and CRN-like proteins are found even in *Batrachochytrium dendrobatidis* (a fungal chytrid pathogen of amphibians) [48] and arbuscular mycorrhizal fungi [49]. In contrast, RxLR effectors have been identified only in plant pathogens of the Peronosporaceae lineage [6]. Second, most CRN effectors are highly expressed. For example, ~50% of CRN-encoding genes are among the top 10% of most highly expressed genes in *P*. *infestans* [25], and the average expression levels of CRN genes in *P*. *sojae* were higher than those of RXLR genes and many housekeeping genes [24]. Third, the CRN effectors are more conserved than the RXLR effectors, and most of those characterized appear to function within the nucleus of host cells [6,22]. These features together suggest that this effector family plays important roles during pathogen—host interactions. The mechanisms by which they act are however largely unknown. Here, we have demonstrated that PsCRN108, a CRN effector from *P*. *sojae*, targets specific host promoters to manipulate plant defense responses.

We demonstrated that PsCRN108 could be translocated into plant cells, where it was targeted to the nuclei. It is a modular protein that contains a conserved N-terminal domain that may mediate translocation of its C-terminal effector domains into host cells [6]. A yeast secretion assay indicated that its predicted signal peptide was functional, and detection of this protein in culture supernatants of *P*. *sojae* lines using Western blotting confirmed that PsCRN108 was secreted by the pathogen into the culture supernatnants, and presumably therefore into apoplastic space during infection. Furthermore, the protein could deliver the C-terminal region of Avr1b into soybean cells, demonstrating that PsCRN108 is translocated into host cells during infection. In this respect, it is similar to other characterized host intracellular effectors from *Phytophthora* pathogens, including *P*. *sojae* Avr1b [5] and Isc1 [9]; and *P*. *infestans* Avr3a [4] and CRN2/8/16 [6]. However, the mechanisms of CRN effector translocation into host cells remain unknown.

PsCRN108 contributes to *P*. *sojae* virulence. Its expression was induced during the early stages of infection, whereas the paralogs *PsCRN112a/b/c* and *PsCRN114* were not. Furthermore, silencing of *PsCRN108* (together with *PsCRN112a/b/c*) led to a dramatic reduction in *P*. *sojae* virulence towards soybean. It has been reported that joint silencing of *PsCRN63/115* also reduced virulence [50]; and that silencing of *Avr3a* or *PITG_03192* compromised the pathogenicity of *P*. *infestans* [7,8]. Despite the fact that the genome of this pathogen contains 100 *CRN* genes and 102 pseudogenes [25], silencing of PsCRN108 and *PsCRN112a/b/c* indicates that these closely-related effectors likely play an essential role during infection. Thus, future screening of diverse soybean cultivars to find *R* genes that can detect this effector to trigger ETI might result in generation of durable resistant cultivars for breeding applications.

We demonstrated that PsCRN108 suppressed plant defenses and increased susceptibility to *Phytophthora* pathogens by inhibiting induction of plant *HSP* expression. Unlike PsCRN63 from *P*. *sojae* and PiCRN8 from *P*. *infestans* that also contribute to virulence [26,27], PsCRN108 did not trigger visible cell death following expression in *N*. *benthamiana* or *A*. *thaliana*. PsCRN63 can disturb plant H_2_O_2_ homeostasis by interacting with plant catalases, while PiCRN8 is a kinase-like effector protein that may interfere with the plant kinase-signaling network [26,27]. Using RNAseq analysis, we found that the expression level of ~45% of *Arabidopsis HSP* genes was significantly reduced in *PsCRN108*- expressing transgenic lines. Suppression of *HSP* expression by PsCRN108 was also observed in *Arabidopsis*, *N*. *benthamiana* and soybean using qRT-PCR. Thus, we inferred that PsCRN108 acts to repress induction of *HSP* gene transcription. Many oomycete RxLR effectors interfere with host gene expression. For example, eight of 33 *P*. *infestans* RxLR effectors suppressed flg22-induced *pFRK1-Luc* activation [51], although the underlying mechanisms were not identified. The *Hyaloperonospora arabidopsidis* effector HaRxL44 induced expression of JA/ET-responsive genes and suppressed expression of SA-responsive genes by interacting with plant mediator subunit 19a [21]. Therefore, manipulating host gene expression is a major mechanism used by oomycete intracellular effectors to interfere with host immune responses.

Importantly, we found that PsCRN108 could specifically target plant *HSP* promoters. Its C-terminal effector domain contains a NLS and an HhH motif. We demonstrated these two motifs are required for PsCRN108 to promote pathogen growth *in planta* and to inhibit induction of *HSP* gene expression. HhH motifs have been documented to have sequence-non-specific DNA-binding activity and are widely distributed in DNA repair and DNA synthesis proteins, such as polymerases [52,53]. The well-defined hydrophobic core of HhH motifs mirrors the symmetry of the DNA-double helix. Thus, we hypothesized that suppression of plant *HSP* gene expression by PsCRN108 might be mediated by DNA-binding activity. Indeed, using ChIP, Y1H and EMSA assays, we found that PsCRN108 could interact with *HSP* promoters. Substitutions of three key residues in the HhH motif abolished its DNA-binding activities and attenuated suppression of *HSP* gene suppression and plant defense, suggesting that associations with plant *HSP* promoters likely mediated PsCRN108-triggered susceptibility. Furthermore, PsCRN108 preferentially targeted promoter regions containing HSE’s in ChIP assays, and mutations in the HSE compromised the ability of PsCRN108 to target HSE-containing promoters in yeast one-hybrid assays. These results indicate that targeting of these promoters by PsCRN108 is sequence-specific and dependent on the HSE. Previous reports have shown that the HhH motif is involved in sequence-non-specific DNA binding [41,52]. One possible explanation is that PsCRN108 may interact with HSFs *in vivo*, gaining their specificity from the HSF. However, the *in vitro* DNA binding data, especially the EMSA assays, suggest that the sequence specificity is intrinsic to PsCRN108. If this is correct, then possibly other domains of PsCRN108 may be responsible for the relative specificity of the interaction of PsCRN108 with HSEs, while the HhH domain may be essential for non-specific stabilization of binding to the double helix. Alternatively, the HhH motif found in PsCRN108 may have evolved sequence specificity. Although PsCRN108 preferentially targeted HSE-enriched regions in the promoters we tested and could directly bind to HSE but not HSEm *in vitro*, we cannot exclude the possibility that PsCRN108 may also bind to other DNA sequences. Future studies addressing the structure of this effector and identification of potential other DNA-binding sites in plants may provide insights into the mechanism of specific interactions of PsCRN108 with HSE’s and other promoter elements.

It has been shown that transcription activator-like (TAL) protein effectors produced by *Xanthomonas* genus, Ankyrin A (AnkA) of *Anaplasma phagocytophilum*, Ank200 and several tandem-repeat containing proteins (TRPs) from *Ehrlichia chaffeensis* may enter host nucleus to directly bind DNA [17,54–56]. *A*. *phagocytophilum* AnkA contains multiple ankyrin repeats that facilitate protein—protein or protein—DNA interactions and was confirmed to bind to the host AT-rich *CYBB* promoter region, leading to silencing of both this gene and other defense genes clustered on chromosomes [56]. *E*. *chaffeensis* TRP120 may activate inflammatory chemokine genes by binding directly to a G+C rich motif in human DNA [55], and Ank200 targets specific sites in promoter regions to reprogram host cell gene expression [54], therefore promoting survival of the pathogen. TAL effectors of the genus *Xanthomonas* are another important group of host DNA-targeting effectors. Recognition of host DNA sequences by TAL effectors is usually specific and mediated by their tandem repeat domains [17]. The above studies suggest that host DNA targeting is an important common strategy utilized by various human and plant bacterial pathogens. Here, we showed that *P*. *sojae*, an oomycete plant pathogen, uses a similar strategy to reprogram host gene expression and that PsCRN108 targeted promoters within the nucleus of plant cells. Therefore, PsCRN108 could represent an oomycete ‘nucleomodulin’, a term proposed by Bierne and Cossart [11].

Based on both *in vivo* and *in vitro* assays, the interaction of PsCRN108 with HSE’s and HSE-containing promoters could suppress the interaction between HSE’s and a heat shock transcription factor (AtHsfA1a), which is an important mediator of abiotic stress responses and a major transcriptional activator of *HSP* genes [44,45,57–59]. The DNA-binding domain (DBD) of heat shock transcription factors is responsible for specific binding of the HSE’s in *HSP* promoters, which is essential for induction of the expression of plant *HSP* genes [38]. *AtHsfA1a* is usually expressed constitutively at low levels; however, its activity is repressed under normal conditions but is activated during stress at multiple levels, including oligomerization, localization, phosphorylation, HSE binding and transcriptional activation during stresses [58]. Both human and plant HSP70s may interact directly with HSE: Hsf1 complexes, resulting in negative feedback regulation of Hsf activity [60,61]. The mechanism by which HSP70 interferes with transcriptional activation of AtHsfA1a is unclear. Here, we found that PsCRN108 might interfere with the regulation of AtHsfA1a in plant cells by suppressing its binding to HSE’s and/or by inhibiting the action of HSE-bound AtHsfA1a. This ability of a pathogen effector to prevent host proteins from binding to their target gene promoters has been reported in only a few bacterial pathogens [16]. For example, the human pathogen *Shigella flexneri* produces outer-membrane protein F (OspF), which interferes with binding of NF-κB to its target gene promoters [62].

Together our results provide evidence that PsCRN108 is a nucleomodulin effector that targets host promoters, possibly by binding host DNA promoter elements directly (Fig 7D). Due to its sequence similarity with PsCRN108, PsCRN112 may also have this property, and make an additional, smaller, contribution to virulence through binding host DNA. Therefore, *P*. *sojae* has evolved a novel strategy to down-regulate the expression of key plant defense genes during infection. To our knowledge, this is the first report that an oomycete effector targets host promoters to manipulate plant defense responses. This study and future work identifying other effectors with similar functions will not only improve our understanding of host transcriptional reprogramming by these pathogens, but also provide opportunities for engineering of disease-resistant crops.

## Materials and Methods

### Microbial strains, plants, and culture conditions

*P*. *sojae* (P6497) and *P*. *capsici* (Pc35) strains were routinely maintained on 2.5% vegetable (V8) juice medium at 25°C in the dark [5]. To prepare zoospores of *P*. *sojae* and *P*. *capsici*, mycelia were cultured in 2.5% liquid V8 juice medium for three days and repeatedly washed with sterilized water at room temperature and incubated in 25°C until sporangia formed. To initiate zoospore release, fresh cold water (4°C) was added and the plates were incubated at 4°C. The concentrations of zoospores were estimated with a hemacytometer. *N*. *benthamiana* plants and light-grown soybeans were grown at 25°C under a 16-h light and 8-h dark photoperiod in an environmentally controlled growth room, while *A*. *thaliana* Columbia-0 (Col-0) was grown at 22°C. Etiolated soybean seedlings were grown at 25°C without light for 4 days before harvesting for inoculation.

### Bioinformatics analysis

The NLS of the PsCRN108 and PsCRN112 proteins was identified using PSORT Prediction (http://psort.hgc.jp/form.html). The signal peptide of the PsCRN108 and PsCRN112 proteins was identified with the SignalP 3.0 server using the signalP HMM prediction algorithm (http://www.cbs.dtu.dk/services/SignalP-3.0/). The HhH motif of PsCRN108 was predicted using the PFAM databases (http://pfam.sanger.ac.uk/). Structure prediction was performed using PSIPRED v. 3.3 (http://bioinf.cs.ucl.ac.uk/psipred/). Predictions of the LFLAK domain, DWL domain and DC domain of the CRN effectors were performed following the methods [6,25], in which a combination of PSI-Blast and a HMM was used to define and identify these domains.

### Yeast signal sequence trap system

The yeast signal sequence trap system assay was performed following the described method [42]. We used the pSUC2T7M13ORI (pSUC2) vector, which carries a truncated invertase gene (*SUC2*) lacking both the initiation Met and signal peptide. The N-terminal regions of PsCRN108 and Avr1b were introduced into pSUC2 to create in-frame fusions to the invertase (all primers and constructs used in this study are listed in S3 and S4 Tables). Briefly, pSUC2-derived plasmids were transformed into yeast, which was plated on CMD-W (minus Trp) plates. The positive clones were then transferred to YPRAA plates to assay invertase secretion. Invertase activity was determined by monitoring the reduction of TTC to the insoluble, red-colored triphenylformazan.

### *P*. *sojae* transformation

The *P*. *sojae* transformation and screening of the putative transformants were carried on as the described methods [5,9,27]. Briefly, the *P*. *sojae* transformants that grew well on V8 medium containing 50 μg/ml G418 were used to select positive transformants. Insertion of the gene into *P*. *sojae* was confirmed by PCR using the genomic DNA of *P*. *sojae* transformants. The silencing efficiency was measured using qRT-PCR. Production of the appropriate proteins by transformants was confirmed by Western blotting.

### Plant manipulation

To generate *PsCRN108*- or *GFP*-transgenic *Arabidopsis* plants, *PsCRN108* (lacking the signal peptide-encoding region) was cloned into pBINGFP2 under the control of the 35S promoter (S4 Table). The resulting constructs and the empty vector were transformed into *Arabidopsis* Col-0 plants separately by *Agrobacterium tumefaciens*-mediated transformation [63]. The transgenic plants were first selected using 25 μg/ml kanamycin and then confirmed by fluorescence microscopy and Western blotting. T2-transgenic lines were used for *P*. *capsici* infection. VIGS of *N*. *benthamiana* was performed as described previously [47]. Briefiy, a fragment of *NbHsp90-1*, which exhibited high sequence similarity to other members of this family, was cloned in an antisense manner into the pTRV2 vector to create a silencing construct (pTRV2-*NbHsp90*). *Agrobacterium* strains harboring the pTRV1 vector and pTRV2:GFP, pTRV2:PDS or pTRV2:*NbHsp90* were mixed in a 1:1 ratio to achieve a final OD_600_ for each strain of 0.5. The co-cultures were then infiltrated into the lower three leaves of 2-week-old plants.

### Plant inoculations

To evaluate functional expression of Avr1b in *P*. *sojae* transformants, seedlings of the soybean cultivars Williams (*rps*) and HARO13 (Harosoy background, *Rps1b*) grown in the light for 7 days. Then the hypocotyls were inoculated with small clumps of mycelia. The number of surviving and dead plants were counted after 2 days to evaluate the ability of the transformants to infect the plants containing *Rps1b* [5,9]. To quantitatively evaluate the overall virulence of *P*. *sojae* transformants, etiolated soybean seedlings of cv. Williams were grown for 4 days, then the hypocotyls were inoculated with approximately 100 *P*. *sojae* zoospores [9]. Lesion lengths were measured after 36 hours, and significant differences compared with the WT were identified using Dunnett’s test. To test the protein expression of *P*. *sojae* transformants over-expressing Avr1b and Avr1bCt fusion proteins during infection, mycelium was used to infect the wounded hypocotyls of soybean plants. The infected hypocotyl tissue samples including mycelium were collected 12 hpi.

For assays of *P*. *capsici* infection of *Arabidopsis*, transgenic *Arabidopsis* leaves were detached and maintained on wet paper in a petri dish. Approximately 100 *P*. *capsici* zoospores were inoculated onto the center of the leaves and incubated in a growth room at 25°C in darkness. Disease development was evaluated using a disease severity index (DSI) on a scale of 0–4. A score of ‘0’ indicates no visible disease symptoms or small necrotic flecks; ‘1’, a lesion diameter less than 0.5 cm; ‘2’, a lesion covering less than 50% of the leaf surface; ‘3’, a lesion covering 50–75% of the leaf surface; and a score of ‘4’ refers to 75–100% collapse of the leaf [64]. The significance of the differences were determined by the Wilcoxon rank sum test.

For assays of *P*. *capsici* infection of gene-silenced *N*. *benthamiana* leaves, two weeks after infiltration of *Agrobacterium* strains harboring vectors for VIGS, the upper leaves corresponding to the photo-bleached leaves in plants separately infected with TRV:*PDS* were detached and used for qRT-PCR analysis and *P*. *capsici* inoculation assays. The lesion areas (cm^2^) were measured at 36 or 48 h after inoculation. Significant differences were identified using *t*-tests. For assays of *P*. *capsici* infection of *N*. *benthamiana* leaves transiently expressing PsCRN108 or control proteins, *Agrobacterium*-infiltrated *N*. *benthamiana* plants were grown in a greenhouse for 48 h, and transformed leaves were then detached for the infection assays. In both cases, the detached leaves were maintained on wet paper in a petri dish, and inoculated with approximately 100 *P*. *capsici* zoospores. The lesion areas (cm^2^) were measured after 24 or 36 h incubation at 25°C. Significant differences compared with leaves expressing GFP were identified using Dunnett’s test.

### Fluorescence microscopy

For fluorescence observations, epidermal cell layers of *N*. *benthamiana* leaves collected 48 h after agroinfiltration or whole *A*. *thaliana* leaves were used. DAPI was used to stain plant cell nuclei. Cells were visualized using confocal microscope (LSM 710; Carl Zeiss) at specific excitation and emission wavelengths (GFP, 488 and 495–530 nm; and DAPI, 405 and 440–470 nm).

### Fluorometric GUS assays

The fluorometric GUS assays were performed as described [65,66]. For the experiments shown in Figs 4D, 4E, 6A and 6B, pAtHSP90.1–2000:GUS, pHSE-35Smin:GUS, pHSEm-35Smin:GUS or p35Smin:GUS were separately co-infiltrated as appropriate with the corresponding protein expression vectors into *N*. *benthamiana* leaves. The leaves were immersed in suspensions of *P*. *capsici* zoospores at a concentration of 10^4^ zoospores/ml 48 h after agroinfiltration. Protein was extracted at 3 hr after zoospore inoculation. p35S min:GUS was used for normalization of the relative GUS activity to calculate a fold change.

### Callose deposition and DAB staining

Etiolated soybean hypocotyls inoculated with *P*. *sojae* and *N*. *benthamiana* and transgenic *A*. *thaliana* leaves inoculated with *P*. *capsici* were stained for callose 10 h after infection following methods described previously [67]. Briefly, the hypocotyls of etiolated soybean, the infiltrated areas of *N*. *benthamiana* leaves, and the transgenic *A*. *thaliana* leaves were immersed in *P*. *sojae* or *P*. *capsici* zoospores suspensions at a concentration of 10^4^ zoospores/ml for 2 hours, then maintained on wet paper in a petri dish for another 8 hours. The inoculated leaves of *N*. *benthamiana* and transgenic *A*. *thaliana* were immersed in 5 ml alcoholic lactophenol [one volume of phenol: glycerol: lactic acid: water (1:1:1:1) and two volumes of ethanol]. The leaves were placed under vacuum for 15 min and then incubated at 65°C until completely cleared of chlorophyll (15–30 min). The leaves were transferred to fresh alcoholic lactophenol for a further 2–24 h. To detect callose, cleared leaves or hypocotyls were rinsed in 50% ethanol, rinsed in water and then stained in 150 mM K_2_HPO_4_ (pH 9.5) containing 0.01% aniline blue. Aniline blue fluorescence was observed in the cleared leaf samples mounted in 25% glycerol by Leica microscopy with filter cube A. The number of callose deposits per microscopic field was counted using Image J software and significant differences were identified by *t*-test in *A*. *thaliana* and by Dunnett’s test in *N*. *benthamiana* and soybean.

Infected *N*. *benthamiana* and *A*. *thaliana* leaves were stained with DAB solution for 8 h in the dark at 10 hpi, and then destained with ethanol before observation by light microscopy. DAB staining was quantified as intensity per unit area using the ImageJ software. Significant differences were identified by *t*-test in *A*. *thaliana* and by Dunnett’s test in *N*. *benthamiana*.

### RNA sequence (RNAseq) profiling

Total RNA was isolated from *PsCRN108*- and *GFP*- transgenic *Arabidopsis* plants infected with *P*. *capsici* for 10 h, followed by Illumina sequencing using a HiSeq2000 to produce 100-bp paired-end data. Two biological replicates were used for each condition. All clean reads were mapped to *Arabidopsis* reference gene sequences using Tophat with default parameters [68]. Based on the length of each gene and the read count uniquely mapped to that gene, gene expression levels were normalized as fragments per kilobase of exon per million fragments mapped (FPKM) using the Cufflinks tool [69]. This method adjusted the number of fragments matching each gene by the total number of mapped fragments and the length of the gene. After that, the Cuffdiff tool was employed to identify gene expression differences with statistical significance (p-value <0.05, FDR-adjusted q-value <0.05; >1.5-fold differential expression change). For functional annotation, BLAST was used to query the sequences against the NCBI NR database using an E-value cut-off of 10^−5^.

### Quantitative RT-PCR analysis

To characterize *PsCRN108* expression during infection, total RNA of *P*. *sojae* P6497 was collected from zoospores and zoospore-infected etiolated soybean seedlings (cv. Williams). Hypocotyls of etiolated soybean seedlings were inoculated with *P*. *sojae* zoospore suspensions at a concentration of 10^4^ zoospores/ml. Samples were collected at the indicated time points. To characterize the silencing efficiency of *PsCRN108* and *PsCRN112* in P6497 transformants, mycelia were collected for RNA extraction. To characterize the *HSP* gene transcript levels in plant tissues, leaves of transgenic *Arabidopsis* plants, *A*. *tumefaciens*-infiltrated *N*. *benthamiana* leaves, or etiolated soybean seedlings with or without pathogen infection were collected. Total RNA was extracted using a NucleoSpin RNA II kit (Invitrogen) following the manufacturer’s instructions and treated with DNase I (TaKaRa) to remove DNA contamination. Approximately 800 ng RNA were used for reverse transcription with oligo(dT) primers. Real-time PCR was performed using an ABI Prism 7500 Fast Real-Time PCR system with SYBR Premix Ex Taq (TaKaRa) according to the manufacturer’s instructions. The comparative threshold cycle (Ct) method was used to determine relative transcript levels through ABI 7500 System Sequence Detection Software. To assess *PsCRN* gene expression during infection, the relative transcript levels were normalized to the *P*. *sojae* zoospore data using the *actin* gene as an internal reference. To characterize the silencing efficiency of *PsCRN108* and *PsCRN112* on P6497 transformants, the relative transcript levels were normalized to the data from the *P*. *sojae* WT strain using the *actin* gene as an internal reference. To characterize *HSP* gene transcript levels in the plants, the relative transcript levels were normalized to the data of GFP-transgenic *Arabidopsis* or *N*. *benthamiana* without pathogen infection or soybean plants without pathogen infection using *Actin2* (*At3G18780*) in *Arabidopsis*, *NbEF1α* in *N*. *benthamiana* and *ACT20* in soybean as internal references.

### Western blot

Protein extraction buffer (50 mM HEPES, 150 mM KCL, 1 mM EDTA, and 0.1% Triton X-100; pH 7.5) supplemented with 1 mM dithiothreitol and protease inhibitor cocktail (Roche) was used for protein extraction from mycelia and plant materials. Ten-day-old *P*. *sojae* culture supernatants were collected and precipitated overnight at 0°C by addition of 70 g (NH4)_2_SO_4_ per 100 ml culture filtrate. The precipitate was collected by centrifugation at 12,000 *g* for 10 min at 4°C and then resuspended in 10 mM Tris-HCl (pH 7.5) and 1 mM EDTA (TE). Protein expression was confirmed by Western blotting following standard procedures. Anti-His primary monoclonal antibody (Sigma-Aldrich) and IRDye 800CW-conjugated goat (polyclonal) anti-mouse IgG secondary antibodies (LI-COR Biosciences) were used to assess *P*. *sojae* transformants; anti-GFP primary monoclonal antibody (Sigma-Aldrich) and IRDye 800CW-conjugated goat (polyclonal) anti-mouse IgG secondary antibodies were used to assess transgenic plants. PVDF membranes were visualized using a scanner (LI-COR Odyssey) with excitation at 700 and 800 nm.

### Chromatin immunoprecipitation (ChIP) assays

*GFP*- and *PsCRN108*-transgenic *Arabidopsis* plants with and without *P*. *capsici* infection were used in this assay. The leaves were immersed in *P*. *capsici* zoospores suspensions at a concentration of 10^4^ zoospores/ml or water for two hours and then maintained on wet paper in a petri dish for another eight hours. ChIP was performed using the GENMED plant chromatin immunoprecipitation kit (Genmed Scientific) as described by the manufacturer. Crude chromatin extract was divided into three parts. One part was saved for use as the input control. The other two parts were used for immunoprecipitation with or without 10 μl anti-GFP antibody (Sigma-Aldrich). After several washes, chromatin crosslinking was reversed, and DNA was purified. q-PCR analysis was performed on immunoprecipitated DNA. Values for the ChIP samples were first normalized to the input control and then divided by the no-antibody control to obtain the fold enrichment values. The primers used to amplify four independent regions of each promoter are listed in S3 Table. For ChIP assay in *N*. *benthamiana*, *A*. *tumefaciens*-infiltrated *N*. *benthamiana* leaves were infected with *P*. *capsici* zoospores, and samples were collected at 10 hpi.

### Yeast one-hybrid (Y1H) assay

Y1H assay was performed using the Matchmaker Gold Yeast One-Hybrid Library Screening System (Clontech) as described in the manufacturer’s instructions. The HSE consensus sequence or its mutated form was synthesized with restriction enzyme overhangs, annealed, and cloned into the pAbAi vector carrying the *AUR1-C* gene. The plasmid was linearized and integrated into the genome of yeast (Y1H Gold). Bait yeast cells were then transformed with the pGADT7-AD plasmid (Invitrogen) containing PsCRN108 or AtHsfA1a. Interactions were identified based on the ability of transformed yeast to grow on -Leu medium in the presence of 200–500 ng ml^-1^ Aureobasidin A.

### Electrophoretic mobility shift assay (EMSA)

MBP:AtHsfA1a and MBP:PsCRN108 mutant proteins were expressed and purified from *E*. *coli* strain Rosetta using the pHMTc construct containing an N-terminal His-tag-coding sequence. 5’ Alex660-labeled HSE primers or HSEm primers were annealed to a double-stranded DNA probe. The DNA fragment from the *AtHSP90*.*1* promoter was end-labeled with Alex660 by PCR amplification using the 5’Alex660-labeled primer. Recombinant fusion protein in binding buffer [25 mM HEPES, pH 8, 10 mM MgCl_2_, 20 mM KCl, 0.1 mM EDTA, 1 mM DTT, 100 ng poly (dI-dC) and 5% glycerol] was mixed with Alex660-labeled DNA and incubated for 3 h at 25°C. The reaction mixtures were electrophoresed in 6% native polyacrylamide gels in 1× Tris-glycine buffer (25 mM Tris-HCl, pH 8.6, 200 mM glycine) at 60 V and 4°C. Gels were directly visualized using a LI-COR Odyssey scanner with excitation at 700 nm.

### DNA-pull down assay

The 5’ biotinylated HSE primers were annealed to a double-stranded DNA probe. MBP-fused proteins and GST-fused proteins were expressed in *E*. *coli* strain Rosetta using the pHMTc construct containing an N-terminal His-coding sequence and pGEX-4T-2 construct respectively. MBP-fused proteins were purified on Ni-NTA agarose (Qiagen) and GST-fused proteins on glutathione sepharose 4B (GE Healthcare) resin according to the manufacturers’ instructions. DNA pull-downs were performed using Streptavidin Agarose Resin (Thermo Scientific) as described in the manufacturer’s instructions. Briefly, 100 fmol biotinylated double-strand HSE were mixed with 150 μg indicated protein in EMSA binding buffer and incubated for 1 h at 25°C. Then streptavidin-agarose were added for another one hour incubation with shaking. After washing of the agarose with binding buffer for three times, the proteins were eluted from the agarose by boiling with SDS-loading buffer, and then were separated on SDS-PAGE and analyzed by western blotting with the corresponding antibodies. For the competitive DNA pull down assay, biotinylated HSE was first incubated with GST:PsCRN108 for one hour and then with streptavidin agarose for one hour. After washing of the unbound protein, the MBP or MBP:AtHsfA1a was added for further incubation of two hours.

### Accession numbers

Sequence data from this article can be found in the GenBank/EMBL data libraries under the following accession numbers: PsCRN108 (KT726855), PsCRN112a (KU051420), PsCRN112b (KU051421), PsCRN112c (KU051422), PsCRN114 (KT726856), GmHsp90A1 (KT726857), NbHsp90-1 (KT726858), NbHsp90-2 (KT726859), NbHsp90-3 (KT726860). From Arabidopsis, they are as follows: AtHSP20 (*AT1G53540*), AtHSP70 (*AT3G12580*), AtHSP90.1 (*AT5G52640*), AtHSP101 (*AT1G74310*), AtHsfA1a (*AT4G17750*), AtHsfA2 (*AT2G26150*).

## Supporting Information

S1 Fig Domain organization and expression patterns of PsCRN108, PsCRN112 and PsCRN114. (A) Predicted domain organization of HhH motif-containing CRN effectors. The relative positions of the signal peptide (SP), LFLAK domain, DWL domain, DC domain, the NLS and the HhH motif are indicated. (B) Sequence alignment of the predicted HhH motifs in *P*. *sojae* CRN effectors and other known HhH motifs. Protein sequences were aligned using Muscle. Identical amino acids are highlighted in blue and less-conserved amino acids in light gray. MutY, *E*. *Coli*, Genbank ID: EGW83408; hOGG1, *Homo sapiens*, Genbank ID: AAH00657; endonuclease III, *E*. *Coli*, Genbank ID: J02857. Asterisks indicate the conserved DNA-binding residues. Structure prediction was performed using PSIPRED v. 3.3 (http://bioinf.cs.ucl.ac.uk/psipred/). (C) *PsCRN* expression profiles during infection. A susceptible soybean cultivar (Williams) was infected with *P*. *sojae* zoospores at the indicated time points (min, minutes post-inoculation; h, hours post-inoculation). Values are the means ± SEM of three independent biological replicates. Common letters indicate values that are not significantly different (P<0.01; Duncan’s multiple range test).
(PDF)

S2 Fig Full length sequence alignments of PsCRN proteins. Identical positions are highlighted in cyan, while polymorphic positions are highlighted in gray and white, with variant amino acids shown in lower case. The specific primer sites for *PsCRN108* are shown above the alignment and the primer sites for *PsCRN112a/b/c* are shown below the alignment. The NLS was identified using PSORT Prediction (http://psort.hgc.jp/form.html). The signal peptide was identified using the signalP HMM prediction algorithm (http://www.cbs.dtu.dk/services/SignalP-3.0/). The HhH motif was predicted using the PFAM databases (http://pfam.sanger.ac.uk/). The LFLAK domain, DWL domain and DC domain of the CRN effectors were obtained from Supplemental File S8 of Haas et al [25].
(PDF)

S3 Fig Silencing of *PsCRN108* and *PsCRN112a/b/c* had no effect on *P*. *sojae* growth. Photographs were taken after 4 days of growth on V8 medium. Plates contain wild type or transgenic *P*. *sojae* lines as described in the text.
(PDF)

S4 Fig Characterization of transgenic *Arabidopsis* plants. (A) Western blot analysis of transgenic *Arabidopsis* lines expressing GFP and GFP:PsCRN108 using an anti-GFP antibody. Protein sizes are in kDa. Ps, Ponceau S staining. (B) Growth of transgenic *Arabidopsis* plants. (C) Nuclear localization of PsCRN108 in transgenic *Arabidopsis* plants. Epidermal cells of transgenic *Arabidopsis* leaves expressing GFP or GFP:PsCRN108 were visualized using confocal microscopy. DAPI staining was used to confirm the location of nuclei. Scale bars = 50 μm (upper panel) and 10 μm (lower panel).
(PDF)

S5 Fig DAB Staining of transgenic *N*. *benthamiana* and *Arabidopsis* leaves. Transgenic *N*. *benthamiana* (A,B) leaves expressing GFP, PsCRN108 or its mutants and *Arabidopsis* leaves (C,D) expressing GFP or PsCRN108 were detached and used for DAB staining 10 h after inoculation with *P*. *capsici* zoospores. Mock treated leaves were used as controls. (A,C) Representative images. (B,D) Quantification of DAB staining as intensity per unit area from 9 leaves per genotype was measured using ImageJ in arbitrary units. Values are the means ± SEM of three independent biological replicates, each of which comprised 3 leaves (**, P<0.01, *t*-test in *Arabidopsis*; **, P<0.01 compared with GFP; Dunnett's test in *N*. *benthamiana*). Scale bars = 100 μm.
(PDF)

S6 Fig Confocal imaging of *N*. *benthamiana* epidermal cells expressing PsCRN108 and the mutants. Photographs were taken 48 h post-infiltration. Values on the y axis indicate relative intensity of GFP fluorescence signal. White lines depict the transects used for the intensity plots. Scale bars = 50 μm (left panel) and 10 μm (right panel). Images in the right hand column are the same as in Fig 3I.
(PDF)

S7 Fig Locations of HSE’s in the promoter regions of plant *HSP* genes. “ATG” is the translational start site for the *HSP* genes. Positions of four variants of known HSE’s are indicated in the promoter region relative to the ATG initiation codon. Positions are shown to scale. Amplicons specific for the S1–4 segments of the five gene promoters analyzed by ChIP (red labels) are indicated by red bars.
(PDF)

S8 Fig Electrophoretic mobility shift assay (EMSA) of PsCRN108 binding to the *AtHSP90*.*1* promoter and to a 19 bp synthetic HSE DNA fragment. (A) SDS-PAGE of fusion proteins purified from *E*. *coli*. MBP:AtHsfA1a and MBP:PsCRN108 variants were expressed and purified from *E*. *coli* strain Rosetta using the pHMTc construct containing an N-terminal His-tag coding sequence. Although adequate amounts of the proteins were produced in *E*. *coli*, both proteins had very low specific activities for DNA binding as judged by the competition assays shown in panel (D). (B-C) Binding of PsCRN108 to *AtHSP90*.*1* promoter and 19 bp HSE. 100 fmol (5 nM) *AtHSP90*.*1*–500 DNA (B), 19 bp HSE or 19 bp HSEm (C) were end-labeled with Alex660 and incubated with MBP (6 μM), MBP:PsCRN108 (6 μM), MBP:PsCRN108-HhHm (6 μM) (DNA:Protein = 1:1200) or AtHsfA1a(1 μM) (DNA:Protein = 1:200) purified from *E*. *coli*. AtHsfA1a was used as the positive control and MBP protein as the negative control. The positions of the protein—DNA complexes are indicated by triangles. 100-fold molar excesses of unlabeled HSE (Un-HSE) or HSEm (Un-HSEm) (500 nM each) were used as the competitors. (D) Dose response of DNA binding and competition measured by EMSA. Increasing amounts of MBP:PsCRN108 (0, 0.25, 0.5, 1, 2, 4, 6 μM) were incubated with 100 fmol (5 nM) Alex660-labeled 19 bp HSE (the first box) for the DNA binding assay; increasing amounts (0- to 100-fold molar excess) of unlabeled 19 bp HSE (the second box) or 19 bp HSEm (the third box) were incubated with 6 μM MBP:PsCRN108 and 100 fmol (5 nM) Alex660-labeled HSE for the competition assays. Similarly, increasing amounts of MBP:AtHsfA1a (0, 0.075, 0.125. 0.25, 0.5, 1, 2 μM) were incubated with 100 fmol (5nM) Alex660-labeled HSE (the fourth box) for the DNA binding assay; increasing amounts (0- to 100-fold molar excess) of unlabeled HSE (the fifth box) and HSEm (the sixth box) were incubated with 1 μM MBP:AtHsfA1a and 100 fmol (5 nM) Alex660-labeled HSE for competition assays. In all cases, the protein-DNA complexes remained in the wells and did not migrate into the gel.
(PDF)

S9 Fig The *NbHsp101* gene family is required for disease resistance in *N*. *benthamiana*. (A) Phenotypes of silencing of *NbHsp90* or *NbHsp101* in *N*. *benthamiana*. VIGS-plants were photographed 14 days after infiltration. Abnormal upper leaves are indicated by arrowheads. (B) Relative transcript levels of *NbHsp101* genes. *N*. *benthamiana* leaves were infiltrated with *Agrobacterium* strains harboring the pTRV1 vector combined with pTRV2:*NbHsp101* or pTRV2:*GFP* (as a negative control). Total RNA samples were extracted 2 weeks after infiltration and subjected to qRT-PCR analysis. Transcriptional levels were calculated by normalization to the levels in *N*. *benthamiana* infiltrated with TRV:*GFP* using the *NbEF1α* gene as an internal reference. Bars represent standard errors from three independent biological replicates (**, P<0.01; *t*-test). (C) Lesion areas of infected leaves at 36 and 48 hpi. Values (cm^2^) are the means ± SEM of three independent biological replicates, each of which comprised five leaves (**, P<0.01, *t*-test). (D) Phenotypes of TRV-*N*. *benthamiana* leaves inoculated with *P*. *capsici*. Representative photographs were taken at 48 hpi with *P*. *capsici* zoospores.
(PDF)

S1 Table Details of differentially expressed genes in transgenic *Arabidopsis* plants. (XLSX)

S2 Table Expression levels of *HSP* genes in transgenic *A*. *thaliana* plants. (XLSX)

S3 Table Oligonucleotides used in the study. (DOCX)

S4 Table Constructs and construction methods used in this study. (DOC)
