# Supplementary figures and images for: An Oomycete CRN Effector Reprograms Expression of Plant HSP Genes by Targeting their Promoters
Source: PLoS Pathog. 2015 Dec 29;11(12):e1005348. doi: 10.1371/journal.ppat.1005348 (PMC4695088; doi:10.1371/journal.ppat.1005348)

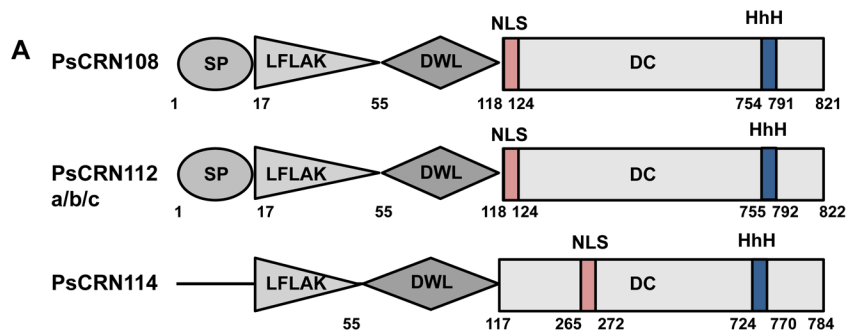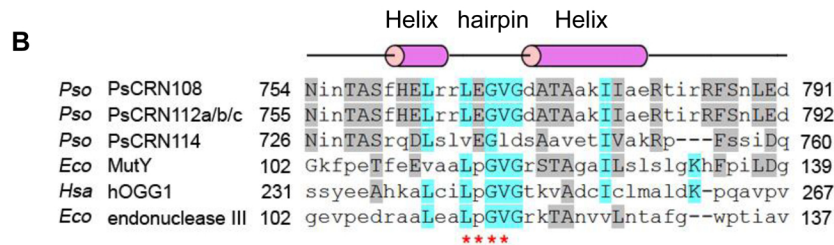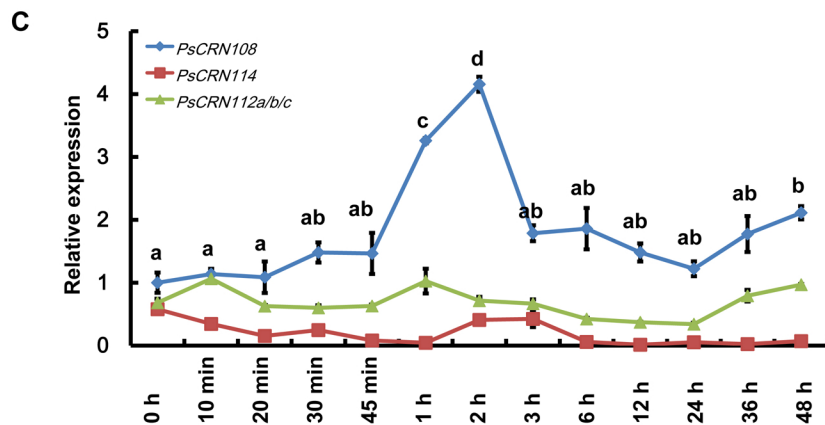

Supplement: S1 Fig — (A) Predicted domain organization of HhH motif-containing CRN effectors. The relative positions of the signal peptide (SP), LFLAK domain, DWL domain, DC domain, the NLS and the HhH motif are indicated. (B) Sequence alignment of the predicted HhH motifs in P. sojae CRN effectors and other known HhH motifs. Protein sequences were aligned using Muscle. Identical amino acids are highlighted in blue and less-conserved amino acids in light gray. MutY, E. Coli, Genbank ID: EGW83408; hOGG1, Homo sapiens, Genbank ID: AAH00657; endonuclease III, E. Coli, Genbank ID: J02857. Asterisks indicate the conserved DNA-binding residues. Structure prediction was performed using PSIPRED v. 3.3 (http://bioinf.cs.ucl.ac.uk/psipred/). (C) PsCRN expression profiles during infection. A susceptible soybean cultivar (Williams) was infected with P. sojae zoospores at the indicated time points (min, minutes post-inoculation; h, hours post-inoculation). Values are the means ± SEM of three independent biological replicates. Common letters indicate values that are not significantly different (P<0.01; Duncan’s multiple range test). (PDF) [file ppat.1005348.s001.pdf]

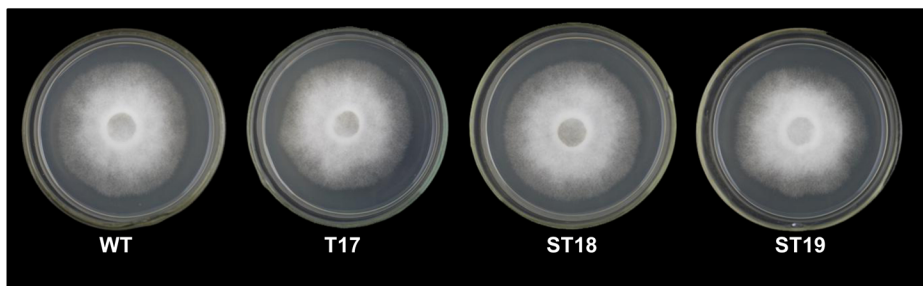

Supplement: S3 Fig — Photographs were taken after 4 days of growth on V8 medium. Plates contain wild type or transgenic P. sojae lines as described in the text. (PDF) [file ppat.1005348.s003.pdf]

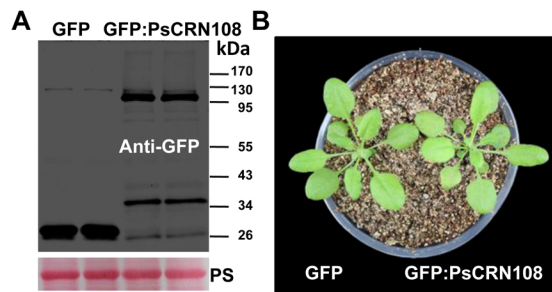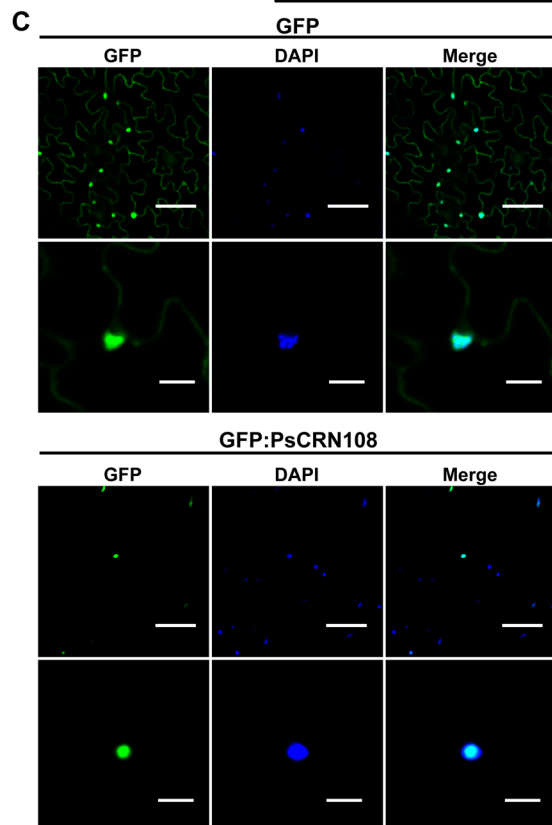

Supplement: S4 Fig — (A) Western blot analysis of transgenic Arabidopsis lines expressing GFP and GFP:PsCRN108 using an anti-GFP antibody. Protein sizes are in kDa. Ps, Ponceau S staining. (B) Growth of transgenic Arabidopsis plants. (C) Nuclear localization of PsCRN108 in transgenic Arabidopsis plants. Epidermal cells of transgenic Arabidopsis leaves expressing GFP or GFP:PsCRN108 were visualized using confocal microscopy. DAPI staining was used to confirm the location of nuclei. Scale bars = 50 μm (upper panel) and 10 μm (lower panel). (PDF) [file ppat.1005348.s004.pdf]

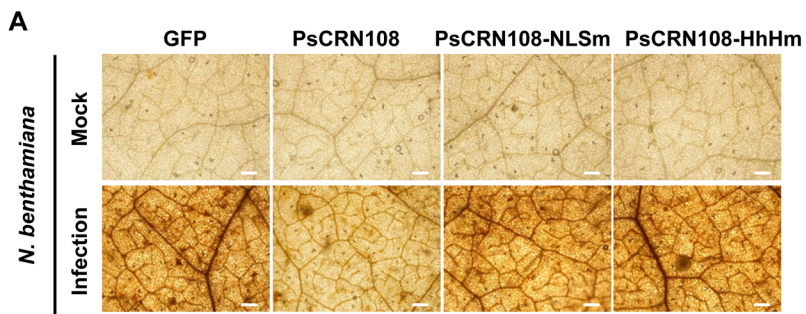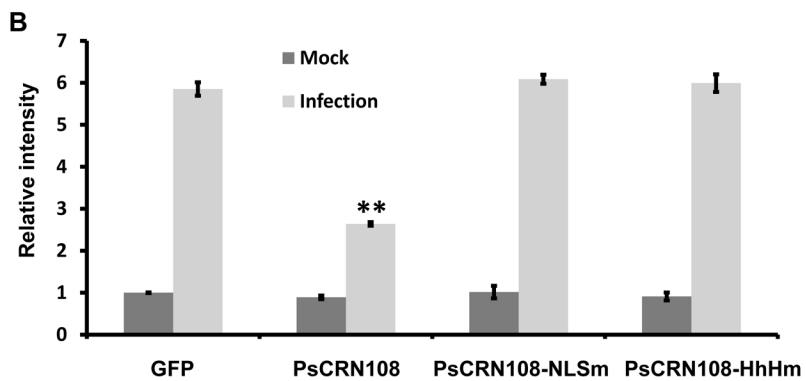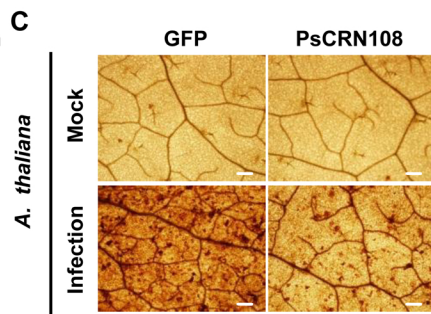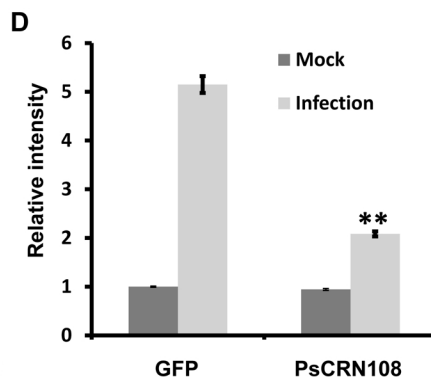

Supplement: S5 Fig — Transgenic N. benthamiana (A,B) leaves expressing GFP, PsCRN108 or its mutants and Arabidopsis leaves (C,D) expressing GFP or PsCRN108 were detached and used for DAB staining 10 h after inoculation with P. capsici zoospores. Mock treated leaves were used as controls. (A,C) Representative images. (B,D) Quantification of DAB staining as intensity per unit area from 9 leaves per genotype was measured using ImageJ in arbitrary units. Values are the means ± SEM of three independent biological replicates, each of which comprised 3 leaves (**, P<0.01, t-test in Arabidopsis; **, P<0.01 compared with GFP; Dunnett's test in N. benthamiana). Scale bars = 100 μm. (PDF) [file ppat.1005348.s005.pdf]

GFP

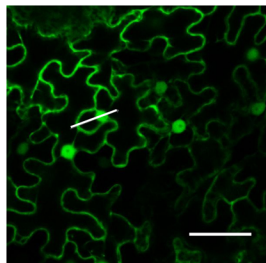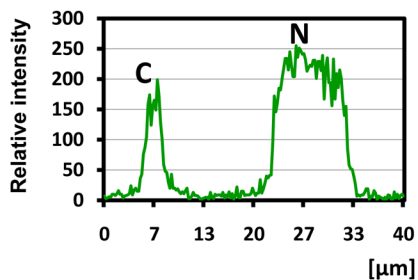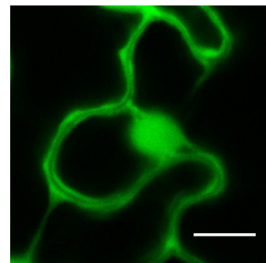

GFP:PsCRN108

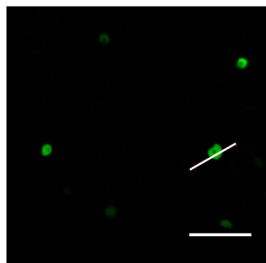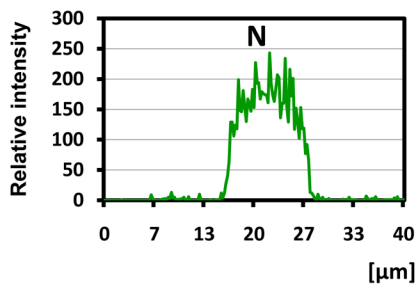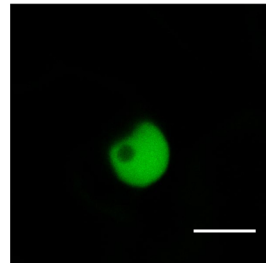

GFP:PsCRN108-  
NLSm

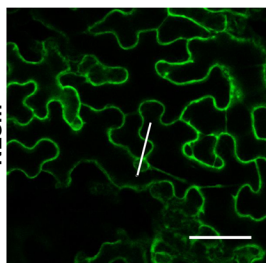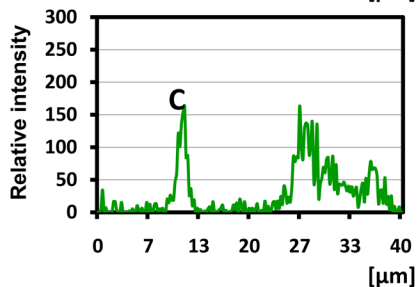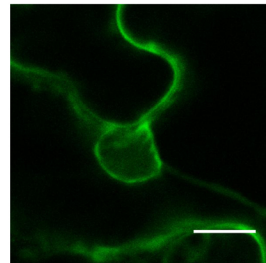

GFP:PsCRN108-  
HhHm

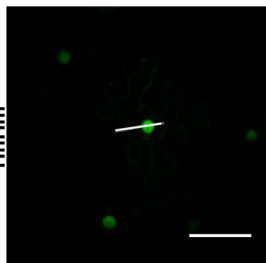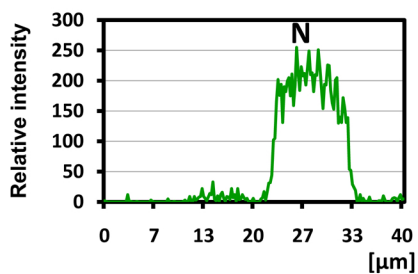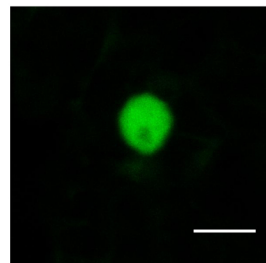

Supplement: S6 Fig — Photographs were taken 48 h post-infiltration. Values on the y axis indicate relative intensity of GFP fluorescence signal. White lines depict the transects used for the intensity plots. Scale bars = 50 μm (left panel) and 10 μm (right panel). Images in the right hand column are the same as in Fig 3I. (PDF) [file ppat.1005348.s006.pdf]

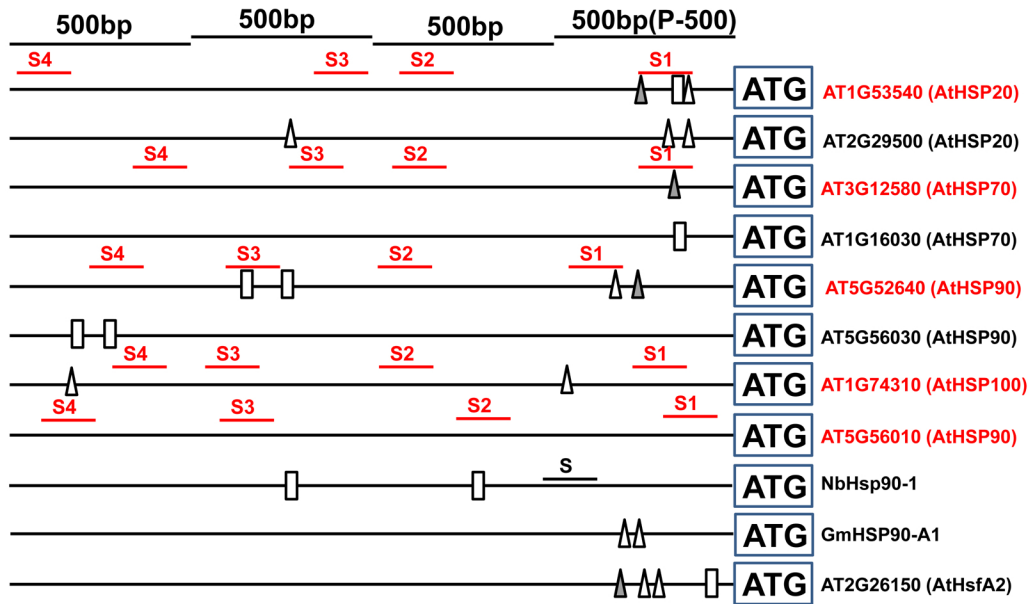

GAA<sub>nn</sub>TTC   
 GAA<sub>nn</sub>TTC<sub>nn</sub>GAA   
 TTC<sub>nn</sub>GAA   
 TTC<sub>nn</sub>GAA<sub>nn</sub>TTC

Supplement: S7 Fig — “ATG” is the translational start site for the HSP genes. Positions of four variants of known HSE’s are indicated in the promoter region relative to the ATG initiation codon. Positions are shown to scale. Amplicons specific for the S1–4 segments of the five gene promoters analyzed by ChIP (red labels) are indicated by red bars. (PDF) [file ppat.1005348.s007.pdf]

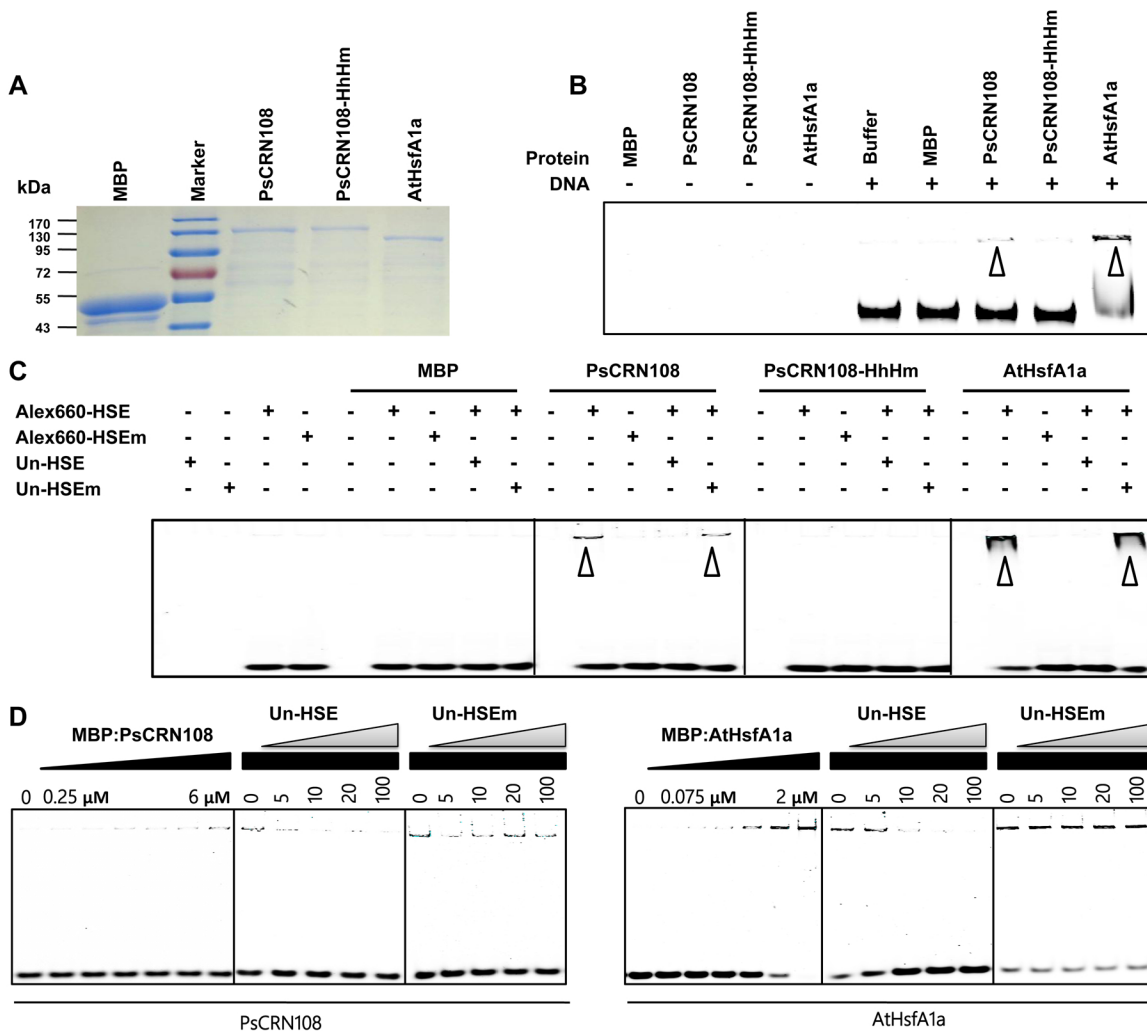

Supplement: S8 Fig — (A) SDS-PAGE of fusion proteins purified from E. coli. MBP:AtHsfA1a and MBP:PsCRN108 variants were expressed and purified from E. coli strain Rosetta using the pHMTc construct containing an N-terminal His-tag coding sequence. Although adequate amounts of the proteins were produced in E. coli, both proteins had very low specific activities for DNA binding as judged by the competition assays shown in panel (D). (B-C) Binding of PsCRN108 to AtHSP90.1 promoter and 19 bp HSE. 100 fmol (5 nM) AtHSP90.1–500 DNA (B), 19 bp HSE or 19 bp HSEm (C) were end-labeled with Alex660 and incubated with MBP (6 μM), MBP:PsCRN108 (6 μM), MBP:PsCRN108-HhHm (6 μM) (DNA:Protein = 1:1200) or AtHsfA1a(1 μM) (DNA:Protein = 1:200) purified from E. coli. AtHsfA1a was used as the positive control and MBP protein as the negative control. The positions of the protein—DNA complexes are indicated by triangles. 100-fold molar excesses of unlabeled HSE (Un-HSE) or HSEm (Un-HSEm) (500 nM each) were used as the competitors. (D) Dose response of DNA binding and competition measured by EMSA. Increasing amounts of MBP:PsCRN108 (0, 0.25, 0.5, 1, 2, 4, 6 μM) were incubated with 100 fmol (5 nM) Alex660-labeled 19 bp HSE (the first box) for the DNA binding assay; increasing amounts (0- to 100-fold molar excess) of unlabeled 19 bp HSE (the second box) or 19 bp HSEm (the third box) were incubated with 6 μM MBP:PsCRN108 and 100 fmol (5 nM) Alex660-labeled HSE for the competition assays. Similarly, increasing amounts of MBP:AtHsfA1a (0, 0.075, 0.125. 0.25, 0.5, 1, 2 μM) were incubated with 100 fmol (5nM) Alex660-labeled HSE (the fourth box) for the DNA binding assay; increasing amounts (0- to 100-fold molar excess) of unlabeled HSE (the fifth box) and HSEm (the sixth box) were incubated with 1 μM MBP:AtHsfA1a and 100 fmol (5 nM) Alex660-labeled HSE for competition assays. In all cases, the protein-DNA complexes remained in the wells and did not migrate into the gel. (PDF) [file ppat.1005348.s008.pdf]

A

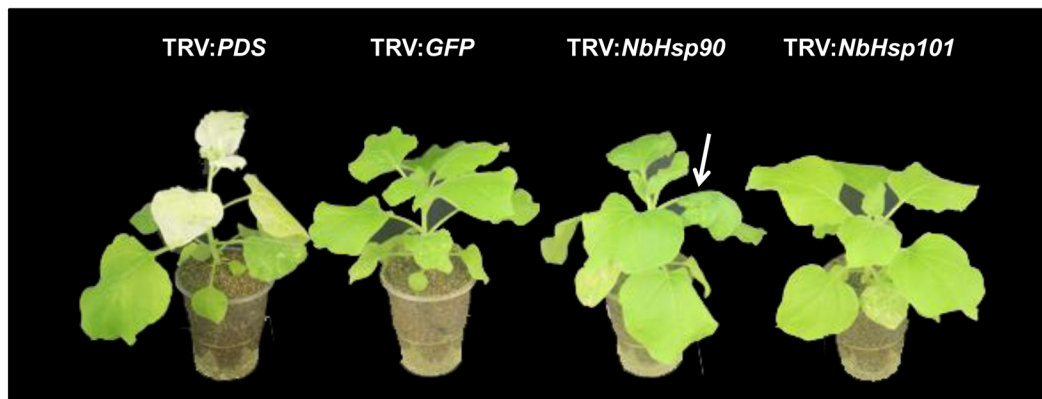

B

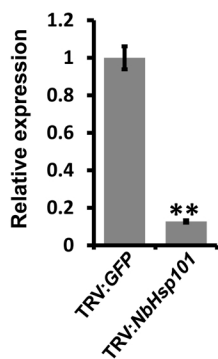

C

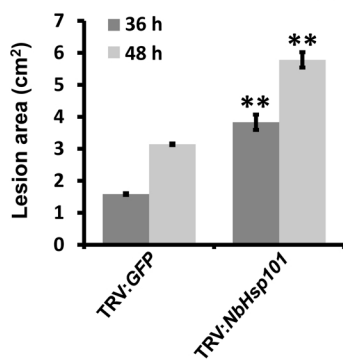

D

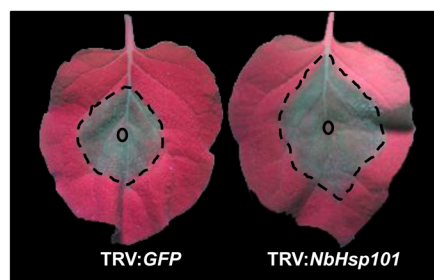

Supplement: S9 Fig — (A) Phenotypes of silencing of NbHsp90 or NbHsp101 in N. benthamiana. VIGS-plants were photographed 14 days after infiltration. Abnormal upper leaves are indicated by arrowheads. (B) Relative transcript levels of NbHsp101 genes. N. benthamiana leaves were infiltrated with Agrobacterium strains harboring the pTRV1 vector combined with pTRV2:NbHsp101 or pTRV2:GFP (as a negative control). Total RNA samples were extracted 2 weeks after infiltration and subjected to qRT-PCR analysis. Transcriptional levels were calculated by normalization to the levels in N. benthamiana infiltrated with TRV:GFP using the NbEF1α gene as an internal reference. Bars represent standard errors from three independent biological replicates (**, P<0.01; t-test). (C) Lesion areas of infected leaves at 36 and 48 hpi. Values (cm2) are the means ± SEM of three independent biological replicates, each of which comprised five leaves (**, P<0.01, t-test). (D) Phenotypes of TRV-N. benthamiana leaves inoculated with P. capsici. Representative photographs were taken at 48 hpi with P. capsici zoospores. (PDF) [file ppat.1005348.s009.pdf]
