# Supplementary material for: An Oomycete CRN Effector Reprograms Expression of Plant HSP Genes by Targeting their Promoters
Source: PLoS Pathog. 2015 Dec 29;11(12):e1005348. doi: 10.1371/journal.ppat.1005348 (PMC4695088; doi:10.1371/journal.ppat.1005348)
Supplement: S2 Fig — Identical positions are highlighted in cyan, while polymorphic positions are highlighted in gray and white, with variant amino acids shown in lower case. The specific primer sites for PsCRN108 are shown above the alignment and the primer sites for PsCRN112a/b/c are shown below the alignment. The NLS was identified using PSORT Prediction (http://psort.hgc.jp/form.html). The signal peptide was identified using the signalP HMM prediction algorithm (http://www.cbs.dtu.dk/services/SignalP-3.0/). The HhH motif was predicted using the PFAM databases (http://pfam.sanger.ac.uk/). The LFLAK domain, DWL domain and DC domain of the CRN effectors were obtained from Supplemental File S8 of Haas et al [25]. (PDF) [file ppat.1005348.s002.pdf]

```
                SP                           LFLAK
PsCRN108    MVKLYCAVVGVAGSAFSVRVDESDTVDDLKDAIKAKKPNDFKDIDADKLELYVAKRDGVW 60
PsCRN112a   MVKLYCAVVGVAGSAFSVRVDESDTVDDLKDAIKAKKPNDFKDIDADKLELYVAKRDGVW 60
PsCRN112b   MVKLYCAVVGVAGSAFSVRVDESDTVDDLKDAIKAKKPNDFKDIDADKLELYVAKRDGVW 60
PsCRN112c   MVKLYCAVVGVAGSAFSVRVDESDTVDDLKksIKAKKPNDlKDIDADKLELYVAKRDGeW 60

                                 DWL
PsCRN108    LTEADVKSGVADITGLVRLEVVRAKLFSVGLSDEVVSEVDAQEEAAGRGPVNVLVVVPMK 120
PsCRN112a   LTEADVKSGVADITGLVRLEVVRAKLFSVGLSDEVVSEVDAQEEAAGRGPVNVLVVVPMK 120
PsCRN112b   LTEADVKSGVADITGLVRLEVVRAKLFSVGLSDEVVSEVDAQEEAAGRGPVNVLVVVPMK 120
PsCRN112c   LTEADVKSGVtDITGLVRLEVVRAKLFSVGLSDEVVSEVDAQEEAAGRGPVNVLVVVPMK 120

            NLS
PsCRN108    KRRVDAGVDEERRFFDTRDFPPLLAPPQRGATVESPEAQWEKLLNSLEWKEPKRLCASSG 180
PsCRN112a   KRRVDAGVDEERRFFDTRDFPPLLAPPQRGATVESPEAQWEKLLNSLEWKEPKRLCASSG 180
PsCRN112b   KRRVDAGVDEERRFFDTRDFPPLLAPPQRGATVESPEAQWEKLLNSLEWKEPKRLCASSG 180
PsCRN112c   KRRVDAGVDEERRFFDTRDFPPLLAPPQRGATVESPEAQWEKLLdSLEWKkPrRLCASSG 180

PsCRN108    QNWPYQGESELAGHLVEPLALHYTAWYLQNEDKQNHAINLVLSGPGTGKSRMLDQMKGLM 240
PsCRN112a   QNWPYQGESELAGHLVEPLALHYTAWYLQNEDKQNHAINLVLSGPGTGKSRMLDQMKGLM 240
PsCRN112b   QNWPYQGESELAGHLVEPLALHYTAWYLQNEDKQNHAINLVLSGPGTGKSRMLDQMKGLM 240
PsCRN112c   QdWPYQGESELAGHLVEPLALHYTAWYLQNEDKQNHAINLVLSGPGTGKSRMLDQMKGLM 240

PsCRN108    CAAAARSNNRKLKERMENAFVFSVTFENGTSATGSLLDRDNPEFDISYRMLYQLSKDrpn 300
PsCRN112a   CAAAARSNNRKLKERMENAFVFSVTFENGTSATGSLLDRDNPEFDISYRMLYQLSKDKKA 300
PsCRN112b   CAAAARSNNRKLKERMENAFVFSVTFENGTSATGSLLDRDNPEFDISYRMLYQLSKDKKA 300
PsCRN112c   CAAAAlSNNRKLKERMENAFVFSVTFENGTSATGSLLDRDNPEFDISYRMLYQLSKDKKA 300
                                                        CTCTCGAAAGACAAAAAAGCG

          GCCAAGACGTTGAAGTCATAT          TTCACATCGTCAATCCCTTT
PsCRN108    WkkFaktLk-syrSLeLdIeaaIgILAKLKGIDDVKKMTVILCVDGLQKLVNDGTKSCDF 359
PsCRN112a   WPVFVDKLRMMCSSLPLRIQIVIDILAKLKGIDDVKKMTVILCVDGLQKLVNDGTKSCDF 360
PsCRN112b   WPVFVDKLRMMCSSLPLRIQIVIDILAKLKGIDDVKKMTVILCVDGLQKLVNDGTKSCDF 360
PsCRN112c   WPVFVDKLRMMCSSLPLRIQIVIDILAKLKGIDDVKKMTVILCVDGLQKLVNDGTKSCDF 360
                     TCACGATCTGAATACGCAAA
PsCRN108    YRVLASVCSFLNSSRAFAVCVCSATiQSPVDKALSDSPQKRVFLVPPPLRGHEVLPTKTR 419
PsCRN112a   YRVLASVCSFLNSSRAFAVCVCSATVQSPVDKALSDSPQKRVFLVPPPLRGHEVLPTKTR 420
PsCRN112b   YRVLASVCSFLNSSRAFAVCVCSATVQSPVDKALSDSPQKRVFLVPPPLRGHEVLPTKTR 420
PsCRN112c   YRVLASVCSFLNSSRAFAVCVCSATVQSPVDlALSDSPQKRVFLVPPPLRGHEVLPTKTR 420

PsCRN108    IEKQLVDDMGGHGRALETLQLFLSHYTKDQLEEMDPTWMFEKlCDALRLQYGDIFASPFF 479
PsCRN112a   IEKQLVDDMGGHGRALETLQLFLSHYTKDQLEEMDPTWMFEKVCDALRLQYGDIFASPFF 480
PsCRN112b   IEKQLVDDMGGHGRALETLQLFLSHYTKDQLEEMDPTWMFEKVCDALRLQYGDIFASPFF 480
PsCRN112c   IEKQLVDDMGGHGRALETLQLFLSHYTKDQLEEMDPTWMFEKVCDALRLQYGDIFASPFF 480

PsCRN108    QDPYNCREVLAAILSRRRYKLFDRIGRTDMTVDCLRSFGLFRWGAEGHLECAFILLVLLM 539
PsCRN112a   QDPYNCREVLAAILSRRRYKLFDRIGRTDMTVDCLRSFGLFRWGAEGHLECAFILLVLLM 540
PsCRN112b   QDPYNCREVLAAILSRRRYKLFDRIGRTDMTVDCLRSFGLFRWGAEGHLECAFILLVLLM 540
PsCRN112c   QDPYNCREVLAAILSRRRYKLFDRIGRTDMTVDCLRSFGLFRWGAEGHLECAFILLVLLM 540
```

```
PsCRN108     QKLPKKLGEVDNFDDHLTRTVLVWQRFEQFVAFYRRVKSIAYCETPVALSSFHAGARFGA 599
PsCRN112a    QKLPKKLGEVDNFDDHLTRTVLVWQRFEQFVAFYRRVKSIAYCETPVALSSFHAGARFGA 600
PsCRN112b    QKLPKKLGEVDNFDDHLTRTVLVWQRFEQFVAFYRRVKSIAYCETPVALSSFHAGARFGA 600
PsCRN112c    QKLPKKLGEVDNFDDHLTRTVLVWQRFEQFVAFYRRVKSIAYCETPVALSgFHAGARFGA 600

PsCRN108     IQDIIITEPTSRTVVEALRQEDTKSSSDDSTCFTNRDGGVKISDMDTIVINGASASAGDL 659
PsCRN112a    IQDIIITEPTSRTVVEALRQEDTKSSSDDSTCFTNRDGGVKISDMDTIVINGASASAGDL 660
PsCRN112b    IQDIIITEPTSRTVVEALRQEDTKSSSDDSTCFTNRDGGVKISDMDTIVINGASASAGDL 660
PsCRN112c    IQDIIITEPTSRTVVEALRQEDTKSSSDDSTCFTNRDGGVKISDMDTIVINGASASAGDL 660

PsCRN108     FMRVQLKVGRQNVQCNEVIQCKLLQTKQKIHEDAYAKERAKAANESSDVFLLVTPAQATE 719
PsCRN112a    FMRVQLKVGRQNVQCNEVIQCKLLQTKQKIHEDAYAKERAKAANESSDVFLLVTPAQATE 720
PsCRN112b    FMRVQLKVGRQNVQCNEVIQCKLLQTKQKIHEDAYAKERAKAANESSDVFLLVTPAQATE 720
PsCRN112c    FMRVQLKVGRQNVQCNEVIQCKLLQTKQKIdEDAYAKERAKAANESSDVFLLVTPAQATE 720

                                                              HhH
PsCRN108     FDLPPRCGLVSANEFGRYFGPFTSRAYRSFLEPPNINTASFHELRRLEGVGDATAAKIIA 779
PsCRN112a    FDLPPRCGLVSANEFGRYFGPFTSRAYRSFLEPPNINTASFHELRRLEGVGDATAAKIIA 780
PsCRN112b    FDLPPRCGLVSANEFGRYFGPFTSRAYRSFLEPPNINTASFHELRRLEGVGDATAAKIIA 780
PsCRN112c    FDLPPRCGLVSANdFGRYFGPFTSRAYRSFLEPPNINTASFHELRRLEGVGDATAAKIIA 780

PsCRN108     ERTIRRFSNLEDALNRLVPSKKGKTAMILSRMHYDDDEADL* 821
PsCRN112a    ERTIRRFSNLEDALNRLVPSKKGKTAMILSRMHYDDDEADL* 822
PsCRN112b    ERTIRRFSNLEDALNRLVPSKKGKTAMILSRMHYDDDEADL* 822
PsCRN112c    ERTIRRFSNLEDALNRLVPSKKGKTAMILSRMHYDDDEADL* 822
```
