# Supplementary material for: An Oomycete CRN Effector Reprograms Expression of Plant HSP Genes by Targeting their Promoters
Source: PLoS Pathog. 2015 Dec 29;11(12):e1005348. doi: 10.1371/journal.ppat.1005348 (PMC4695088; doi:10.1371/journal.ppat.1005348)
Supplement: S3 Table — (DOCX) [file ppat.1005348.s012.docx]

**Table S3. Oligonucleotides used in the study.**

| **No** | **Name** | **Sequences (5' to 3’)** | **Purpose** |
| --- | --- | --- | --- |
| 1 | actinAF2 | ACTGCACCTTCCAGACCATC | qRT-PCR analysis of reference gene *Actin* in *P.sojae* |
| 2 | actinAR2 | [CCACCACCTTGATCTTCATG](mailto:synsh@invitrogen.com) |  |
| 3 | PsCRN108-RT-F | GCCAAGACGTTGAAGTCATAT | qRT-PCR analysis of*PsCRN108* in *P.sojae* |
| 4 | PsCRN108-RT-R | TTCACATCGTCAATCCCTTT |  |
| 5 | PsCRN112-RT-F | CTCTCGAAAGACAAAAAAGCG | qRT-PCR analysis of*PsCRN112* in *P.sojae* |
| 6 | PsCRN112-RT-R | TCACGATCTGAATACGCAAA |  |
| 7 | PsCRN114-RT-F | CGTATCAAGGAGCGTCTG | qRT-PCR analysis of*PsCRN114* in *P.sojae* |
| 8 | PsCRN114-RT-R | CGTCGTGGTTGTTCTCTT |  |
| 9 | pSUC2-Avr1b-F | ggaattcATGCGTCTATCTTTTGTGC | Insertion of Avr1b signal peptide into pSUC2 vector |
| 10 | pSUC2-Avr1b-R | ccgctcgag CTCAGTTGCGTTGCAGGTCA |  |
| 11 | pSUC2-PsCRN108-F | ggaattcATGGTGAAGCTCTACTGTG | Insertion of PsCRN108 signal peptide into pSUC2 vector |
| 12 | pSUC2-PsCRN108-R | ccgctcgagATCTACTCGCACCGAGAAC |  |
| 13 | avr1b-F | ggaattcacaacaATGCGTCTATCTTTTGTGC | Insertion of *Avr1b:RFP fusion* gene into *P. sojae* transformation vector |
| 14 | avr1b:RFP-R1 | GTCCTCGGAGGAGGCCATGCTCTGATACCGGTGAAA |  |
| 15 | RFP-R2 | gctctagaTTAGTGATGGTGATGGTGATGGGCGCCGGTGGAGTGGCG |  |
| 16 | avr1bCt-F | ggaattcacaacaATGACCTTCAGCGTGACTGACCTGT | Insertion of *Avr1bCt:RFP* fusion gene into *P. sojae* transformation vector |
| 17 | PsCRN108-Spe1-F | gactagtacaacaATGGTGAAGCTCTACTGTG | Insertion of *PsCRN108:Avr1bCt* fusion gene into *P. sojae* transformation vector |
| 18 | PsCRN108:Avr1b-R1 | GGTCAGTCACGCTGAAGGTCAGATCAGCCTCATCGTCA |  |
| 19 | avr1b-R | gctctagaTCAGTGATGGTGATGGTGATG |  |
| 20 | PsCRN108-Xba I-F | gctctagaATGGTGAAGCTCTACTGTG | Insertion of *PsCRN108* segment into *P. sojae* transformation vector |
| 21 | PsCRN108-EcoR I-R | ggaattcCTCTGGTGATTCCACAGTC |  |
| 22 | pBIN2-PsCRN108-F | gggATGGTGCGAGTAGATGAGAGC | Insertion of *PsCRN108* gene into pBinGFP2 vector |
| 23 | pBIN2-PsCRN108-R | gctctagaTTACAGATCAGCCTCATCG |  |
| 24 | pBIN2-PsCRN108-mNLS-R1 | CACTGCAGCTGCAGCCATCGGAACCACCACCAGCA | Insertion of *PsCRN108-NLSm* into pBinGFP2 vector |
| 25 | pBIN2-PsCRN108-mNLS-F2 | CCGATGGCTGCAGCTGCAGTGGATGCGGGAGTGGACGAGGA |  |
| 26 | pBIN2-PsCRN108-HhHm-R1 | CCTGCAGCCTCAGCTCGACGTAACTCATGGAATGA | Insertion of *PsCRN108-HhHm* into pBinGFP2 vector |
| 27 | pBIN2-PsCRN108-HhHm-F2 | TCGAGCTGAGGCTGCAGGTGACGCAACTGCCGCGAAG |  |
| 28 | Ara-Actin2-F | ACCGTATGAGCAAAGAAAT | qRT-PCR analysis of reference gene *Actin2* (At3g18780) in *A. thaliana* |
| 29 | Ara-Actin2-R | CTGAGGGAAGCAAGAATG |  |
| 30 | AT2G29500-RT-F | GGAACTAACATCATCATCAC | qRT-PCR analysis of *A. thaliana* gene AT2G29500 |
| 31 | AT2G29500-RT-R | ATCCTCCTCAATCTCAACT |  |
| 32 | AT1G16030-RT-F | CTTGATGTTGCTCCGCTTA | qRT-PCR analysis of *A. thaliana* gene AT1G16030 |
| 33 | AT1G16030-RT-R | CGCCTGGTTGATTGTCTG |  |
| 34 | AT1G74310-RT-F | GTGCGAATCCAAGACAGA | qRT-PCR analysis of *A. thaliana* gene AT1G74310 |
| 35 | AT1G74310-RT-R | CACAAGCCTCATCAACCA |  |
| 36 | AT1G53540-RT-F | TGAGGAAGGAAGAGGTGAA | qRT-PCR analysis of *A. thaliana* gene AT1G53540 |
| 37 | AT1G53540-RT-R | GGTGCCACTTGTCATTCT |  |
| 38 | AT5G52640-RT-F | AGTGACGATGAGGATGAAG | qRT-PCR analysis of *A. thaliana* gene AT5G52640 |
| 39 | AT5G52640-RT-R | TTCTGCTTGTTGATGAGTTC |  |
| 40 | AT5G56030-RT-F | GACGCAGACAAGAACGAC | qRT-PCR analysis of *A. thaliana* gene AT5G12020 |
| 41 | AT5G56030-RT-R | CCGAAAGTGTTTGGCTCA |  |
| 42 | AT3G12580-RT-F | TGAGGAGGCTTAGGACAG | qRT-PCR analysis of *A. thaliana* gene AT3G12580 |
| 43 | AT3G12580-RT-R | CGAGTGATGGTAGTGTAGAA |  |
| 44 | NbEF1ɑ-QF | AGAGGCCCTCAGACAAAC | qRT-PCR analysis of reference gene *EF1ɑ in N. benthamiana* |
| 45 | NbEF1ɑ-QR | TAGGTCCAAAGGTCACAA |  |
| 46 | NbHSP90-1-RT-F | ACAAGTTCTACGAGGCATT | qRT-PCR analysis of *N. benthamiana NbHSP90*-1 gene |
| 47 | NbHSP90-1-RT-R | GTGGTATCTCAGCAGTTCA |  |
| 48 | NbHSP90-2-RT-F | GATGAGAGTGAAGATGAGAAG | qRT-PCR analysis of *N. benthamiana NbHSP90*-2 gene |
| 49 | NbHSP90-2-RT-R | TAACCAAGCAGCAAGGAG |  |
| 50 | NbHSP90-3-RT-F: | GACCGTGTTGTGGATTCT | qRT-PCR analysis of *N. benthamiana NbHSP90*-3 gene |
| 51 | NbHSP90-3-RT-R | GAGCCTGTGCCTTCATAA |  |
| 52 | ACT20-F | TCGAGGCACCTAATTCTTGG | qRT-PCR analysis of reference gene *ACT20*in *G. max* |
| 53 | ACT20-R | GTGTCTGGATTGGTGGCTCT |  |
| 54 | GmHSP90A1-RT-F | ACCTTGTGTTGCTTCTCTT | qRT-PCR analysis of *G. max GmHSP90A1* gene |
| 55 | GmHSP90A1-RT-R | CGCCATTGTCATCCTCAT |  |
| 56 | TRV2-NbHSP90-F | gggATGGACAATTGCGAAGAAC | Insertion of conserved*NbHsp90*segment into pTRV2 vector for VIGS of *NbHsp90* genes in *N. benthamiana* |
| 57 | TRV2-NbHSP90-R | GCTCTAGAGTCGTATTCTTTTAATTGCCCG |  |
| 58 | AtHSP90.1-P2000-F | ggggtaccATTAATTTCTACTCCAAATCACAGCTTCCG | Insertion of *AtHSP90.1* gene promoter into pMDC162 vector |
| 59 | AtHSP90.1-R | ggcgcgccTCGCAACGAACTTTGATTCAAC |  |
| 60 | pMDC-HSE-F | cCCAGAAGCTTCCAGAAGCCa | Insertion of HSE sequence into pMDC162 vector |
| 61 | pMDC-HSE-R | gatctGGCTTCTGGAAGCTTCTGGggtac |  |
| 62 | pMDC-HSEm-F | cCCATAAGCTTACATAAGCCa | Insertion of HSEm sequence into pMDC162 vector |
| 63 | pMDC-HSEm-R | gatctGGCTTATGTAAGCTTATGGggtac |  |
| 64 | HSE-F | cCCAGAAGCTTCCAGAAGCCc | Insertion of HSE sequence into pAbAi vector |
| 65 | HSE-R | tcgagGGCTTCTGGAAGCTTCTGGggtac |  |
| 66 | HSEm-F | cCCATAAGCTTACATAAGCCc | for insertion of HSEm sequence into pAbAi vector |
| 67 | HSEm-R | tcgagGGCTTATGTAAGCTTATGGggtac |  |
| 68 | pAbAi-AtHsp90.1-F | ggggtaccTTCATGTCCGTATGTACGAGAC | Insertion of *AtHSP90.1* gene promoter sequence into pAbAi vector |
| 69 | pAbAi-AtHsp90.1-R | ccgctcgagTCGCAACGAACTTTGATTCAAC |  |
| 70 | pGAD-PsCRN108-F | ggaattccatATGCGAGTAGATGAGAGC | Insertion of *PsCRN108* gene into pGAD vector |
| 71 | pGAD-PsCRN108-R | cgggatccTTACAGATCAGCCTCATCG |  |
| 72 | CRN108-BamHI-F | cgggatccATGGTGCGAGTAGATGAGAGC | Insertion of *PsCRN108* gene into pGEX4T vector |
| 73 | CRN108-SmaI-R | gggTTACAGATCAGCCTCATCGTCA |  |
| 74 | AtHsfA1a-XbaI-F | gctctagaATGTTTGTAAATTTCAAATACTTC | Insertion of *AtHsfA1a* gene into pHTMc vector |
| 75 | AtHsfA1a-PstI-R | aactgcagCTAGTGTTCTGTTTCTGATGTG |  |
| 76 | CRN108-BamHI-F | cgggatccATGGTGCGAGTAGATGAGAGC | Insertion of *PsCRN108* gene into pHTMc vector |
| 77 | pBIN-GFP-PsCRN108- Xba I -R | gctctagaTTACAGATCAGCCTCATCG |  |
| 78 | Ara-HSP20-S1-F | CCTGAGAAGTCTGGTAACG | CHIP-PCR analysis of *AtHSP20* promoter |
| 79 | Ara-HSP20-S1-R | GGAGAGAGTATGGAAAGTAAGA |  |
| 80 | Ara-HSP20-S2-F | GTATGCTTCAACTGATTCAC |  |
| 81 | Ara-HSP20-S2-R | ACAGATGGTTACTGATGAGA |  |
| 82 | Ara-HSP20-S3-F | GTGGATCATTGCTAGTTATTGG |  |
| 83 | Ara-HSP20-S3-R | GATTGGACGCAGTGTAGAG |  |
| 84 | Ara-HSP20-S4-F | CGGTGACGTTGTATTGGT |  |
| 85 | Ara-HSP20-S4-R | AGTGAGTCTATCGCCTTCT |  |
| 86 | Ara-HSP70-S1-F | TAAGATCCAACGGCTGAGA | CHIP-PCR analysis of *AtHSP70* promoter |
| 87 | Ara-HSP70-S1-R | GAGGAAGAGAAGGCAGAGA |  |
| 88 | Ara-HSP70-S2-F | ACTTTCTTGGCATCTTCATC |  |
| 89 | Ara-HSP70-S2-R | ACTCAAGTGGCTTCGTATT |  |
| 90 | Ara-HSP70-S3-F | CTTGTAGTCTCTTCTGTGGA |  |
| 91 | Ara-HSP70-S3-R | TGGAATCAGTGTTGCCTTA |  |
| 92 | Ara-HSP70-S4-F | GACTCTTAGCTGTTCTTCTC |  |
| 93 | Ara-HSP70-S4-R | GTGACACAATCCGACAAG |  |
| 94 | Ara-HSP90-S1-F | TTGAAGATGGCAAGTGTTCT | CHIP-PCR analysis of *AtHSP90* gene promoter |
| 95 | Ara-HSP90-S1-R | CCGAATAGGTCTGGCAAAG |  |
| 96 | Ara-HSP90-S2-F | AGTCCACAAGAACACAGAG |  |
| 97 | Ara-HSP90-S2-R | GCATAAGCATTGGATAACGA |  |
| 98 | Ara-HSP90-S3-F | AAGTGTGGTGTTCCTGAAG |  |
| 99 | Ara-HSP90-S3-R | GCATACGCCTTGAGCATA |  |
| 100 | Ara-HSP90-S4-F | TTGAGTCGTTGTGATATTGG |  |
| 101 | Ara-HSP90-S4-R | GAACATCTTCCTCGCATAA |  |
| 102 | Ara-HSP101-S1-F | TACCGCTATAATCTGCTTGA | CHIP-PCR analysis of *AtHSP101* gene promoter |
| 103 | Ara-HSP101-S1-R | GGTTTGTTTCGTTGAGTTTC |  |
| 104 | Ara-HSP101-S2-F | AAGCAAGATTGGTCTGAGA |  |
| 105 | Ara-HSP101-S2-R | CCTATCCTCTGTTCTTCCTC |  |
| 106 | Ara-HSP101-S3-F | CACTCTGATGCTATTGCTTGTA |  |
| 107 | Ara-HSP101-S3-R | TTCAAATGACGATGGGTGGTA |  |
| 108 | Ara-HSP101-S4-F | CGATACGACCATTACCAA |  |
| 109 | Ara-HSP101-S4-R | GGTGAAGGTGTTGAGATT |  |
| 110 | Ne-F4 | CCAGCCTTGGTCATCCCA | CHIP-PCR analysis of *AtHSP101* gene promoter*AT5G56010*gene promoter |
| 111 | Ne-R4 | GCAAGCTCGATGGTCAGC |  |
| 112 | Ne-F3 | TCGGAGGAGTTACTGATG |  |
| 113 | Ne-R3 | ATAATGGCGGACGCAGAG |  |
| 114 | Ne-F2 | CCCACAAACCCCACGCTT |  |
| 115 | Ne-R2 | TTCCTGTTTACAGATTGG |  |
| 116 | Ne-F1 | CCTTATAGGTTCCCAGTC |  |
| 117 | Ne-R1 | CTAAGAGCTAGGGTTTAT |  |
| 118 | NbHsp90-1-PF | CTCTAACAATACGGAAAT | CHIP-PCR analysis of *NbHsp90-1* gene promoter |
| 119 | NbHsp90-1-PR | ACCAAAATCTAAGAAAGG |  |
| 120 | pBIN2-PsCRN108-F | gggATGGTGCGAGTAGATGAGAGC | Insertion of *PsCRN108* gene into pBin-HA vector |
| 121 | pBIN-HA-PsCRN108-R | cgggatccCAGATCAGCCTCATCGTCA |  |

Notes: Uppercase letters indicate bases that match the initial template. Lower case letters indicate mutations or 5’ extensions that do not match the initial template. Restriction sites introduced into the amplicon are underlined.
