# Supplementary material for: An Oomycete CRN Effector Reprograms Expression of Plant HSP Genes by Targeting their Promoters
Source: PLoS Pathog. 2015 Dec 29;11(12):e1005348. doi: 10.1371/journal.ppat.1005348 (PMC4695088; doi:10.1371/journal.ppat.1005348)
Supplement: S4 Table — (DOC) [file ppat.1005348.s013.doc]

**Table S4. Constructs and construction** methods used in the study

| No | Plasmid Name | Sources | Construct and purpose | Plasmid used in figures |
| --- | --- | --- | --- | --- |
| 1 | pSUC2.Avr1b | pSUC2 | Containing N terminal of Avr1b [the predicted signal peptide sequences and the following two amino acids (1-23)] fused with invertase for overexpression in yeast. | Fig 1A |
| 2 | pSUC2.PsCRN108 | pSUC2 | Containing N terminal of PsCRN108 [the predicted signal peptide sequences and the following four amino acids (1-21)] fused with invertase for overexpression in yeast. | Fig 1A |
| 3 | pTOR.Avr1b:mRFP | pTOR | Containing Avr1b fused with mRFP for overexpression in *P. sojae*. | Fig 1B, C |
| 4 | pTOR.Avr1bCt:mRFP | pTOR | Containing Avr1b C termini (Avr1bCt aa 66–138, lacking the predicted signal peptide and host translocation domain) fused with mRFP, for overexpression in *P. sojae*. | Fig 1B, C |
| 5 | pTOR.PsCRN108:Avr1bCt | pTOR | Containing full length of PsCRN108 fused with Avr1b C termini for overexpression in *P. sojae*. | Fig 1B, C |
| 6 | pTOR.PsCRN108 | pTOR | Containing partial sequence of *PsCRN108* gene (471 bp) in antisense for gene silencing in *P. sojae*. | Fig 2 and Fig S2 |
| 7 | pBinGFP2.PsCRN108 | pBinGFP2 | Containing mature PsCRN108 ( without signal peptide/ aa 2-17) fused to the C-terminal of GFP for expression in plants*.* | Fig 1E, F; Fig 3; Fig 4 A, B, C; Fig S3; Fig S4 and Fig S5 |
| 8 | pBinGFP2.PsCRN108-NLSm | pBinGFP2 | Containing mature PsCRN108 with a mutated NLS fused to the C-terminal of GFP for expression in plants. | Fig 1E; Fig 3 and Fig S5 |
| 9 | pBinGFP2.PsCRN108-HhHm | pBinGFP2 | Containing mature PsCRN108 with a mutated HhH motif fused to the C-terminal of GFP for expression in plants. | Fig 1E; Fig 3 and Fig S5 |
| 10 | pMDC162.AtHSP90.1  -P2000 | pMDC162 | A 2000 bp promoter region of *AtHsp90.1* was inserted into pMDC162 vector at upstream of *GUS* gene for expression in plants. | Fig 4D, E |
| 11 | pMDC162.HSE | pMDC162 | Conserved HSE sequence was inserted to pMDC162 vector at upstream of *GUS* gene for expression in plants. | Fig 6A, B |
| 12 | pMDC162.HSEm | pMDC162 | Mutated HSE sequence was inserted to pMDC162 vector upstream of *GUS* gene expression in plants. | Fig 6A, B |
| 13 | pAbAi.AtHsp90.1 | pAbAi | A 500 bp promoter region of *AtHsp90.1* was inserted to pApAi vector upstream of the yeast iso-1-cytochrome C minimal promoter and the *AUR1-C* gene for transformation to yeast. | Fig 5B |
| 14 | pAbAi.HSE | pAbAi | Conserved HSE sequence was inserted to pApAi vector upstream of the yeast iso-1-cytochrome C minimal promoter and the AUR1-C gene for transformation to yeast. | Fig 5B |
| 15 | pAbAi.HSEm | pAbAi | Mutated HSE sequence was inserted to pApAi vector upstream of the yeast iso-1-cytochrome C minimal promoter and the *AUR1-C* gene for transformation to yeast. | Fig 5B |
| 16 | pGAD.PsCRN108 | pGAD | Containing PsCRN108 fused to a GAL4 activation domain for overexpression in yeast. | Fig 5B |
| 17 | pGAD.PsCRN108-HhHm | pGAD | Containing PsCRN108 with a mutated HhH motif fused to a GAL4 activation domain for overexpression in yeast. | Fig 5B |
| 18 | pGAD.AtHsfA1a | pGAD | Containing AtHsfA1a fused to a GAL4 activation domain for overexpression in yeast. | Fig 5B |
| 19 | pHMTc.PsCRN108 | pHMTc | Containing PsCRN108 fused to the C-terminal of MBP for expression in Rosetta. | Fig 6C, D and Fig S7 |
| 20 | pHMTc.PsCRN108-HhHm | pHMTc | Containing PsCRN108 with a mutated HhH motif fused to the C-terminal of MBP for expression in Rosetta. | Fig 6C, D and Fig S7 |
| 21 | pHMTc.AtHsfA1a | pHMTc | Containing *AtHsfA1a* gene fused to the C-terminal of MBP for expression in Rosetta. | Fig 6C, D and Fig S7 |
| 22 | pGEX4T.PsCRN108 | pGEX4T-2 | Containing *PsCRN108* gene fused to the C-terminal of GST for expression in Rosetta. | Fig 6C, D |
| 23 | pBin. PsCRN108.HA | pBIN-HA | Containing PsCRN108 fused to the N-terminal of HA for transient expression in *N. benthamiana.* | Fig 6E, F |
| 24 | pBin. PsCRN108-HhHm.HA | pBIN-HA | Containing PsCRN108 with a mutated HhH motif fused to the N-terminal of HA for transient expression in *N. benthamiana.* | Fig 6E, F |
| 25 | pTRV2.NbHsp90 | pTRV2 | Containing part sequence of *NbHsp90*conserved segment for VIGS in *N. benthamiana.* | Fig 7 and Fig S8 |
| 26 | pTRV2.NbHsp101 | pTRV2 | Containing part sequence of *NbHsp101*for VIGS in *N. benthamiana.* | Fig 7 and Fig S8 |
